# Solar Farms as Potential Future Refuges for Bumblebees

**DOI:** 10.1111/gcb.70537

**Published:** 2025-10-08

**Authors:** Hollie Blaydes, Emma Gardner, J. Duncan Whyatt, Simon G. Potts, Robert Dunford‐Brown, John W. Redhead, Alona Armstrong

**Affiliations:** ^1^ Lancaster Environment Centre Lancaster University Lancaster UK; ^2^ UK Centre for Ecology and Hydrology Wallingford UK; ^3^ Centre for Agri‐Environmental Research, School of Agriculture, Policy and Development University of Reading Reading UK; ^4^ Energy Lancaster, Science and Technology Building Lancaster University Lancaster UK

**Keywords:** habitat management, land‐use change, pollinators, renewable energy, solar farm

## Abstract

Solar farms offer an opportunity for habitat creation for wildlife, including insect pollinators, potentially simultaneously contributing to both low‐carbon energy and nature recovery. However, it is unknown whether cobenefits would persist under future land‐use change given that habitat value is context dependent. For the 1042 operational solar farms in Great Britain, we predict their ability to support bumblebee populations (both inside and outside the solar farm) under three different socioeconomic futures. These futures represent alternative 1 km scale landcover projections for the year 2050 with accompanying narratives. We downscale these to 10 m resolution, spatially allocating crop rotations, agri‐environment interventions and other habitat features consistent with the scenario narratives, to realistically represent fine‐scale landscape elements of relevance to bumblebee populations. We then input these detailed maps into a sophisticated process‐based model that simulates bumblebee foraging and population dynamics, enabling us to predict bumblebee density in and around Great Britain's solar farms, accounting for the effects of their changed habitat context and configuration in these different future scenarios. We isolate the drivers of bumblebee density change across scenarios and scales and show that solar farm management was the main driver of bumblebee density within solar farms, with ~120% higher densities inside florally enhanced compared to turf grass solar farms, although the exact figure was influenced by wider landcover changes. In foraging zones immediately surrounding solar farms, landscape changes had a greater impact on bumblebee densities, suggesting a single solar farm in isolation generally did not counteract the influence of wider land‐use changes expected under future scenarios. In addition to providing insights into the potential future value of pollinator habitat on solar farms, our methodology demonstrates how combining process‐based modelling with landcover projections that are downscaled to ecologically relevant resolutions can be used to better assess future effectiveness of habitat interventions. This represents a step change in our ability to account for species' interactions with socioeconomically driven futures, which can be extended and applied to other taxa and land‐use interventions.

## Graphical abstract

Solar farms offer an opportunity for habitat creation for insect pollinators, but it is unknown whether cobenefits would persist under future land‐use changes. For solar farms across Great Britain, we predict their ability to support bumblebees in 2050 under different land‐use and management scenarios. We show that despite changing bumblebee numbers in future landscapes, florally enhanced solar farms can still generally support greater bumblebee populations across different futures.

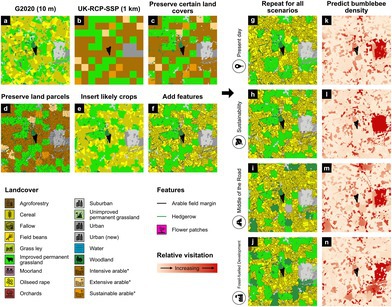

## 1 Introduction

Countries across the world are decarbonising their energy systems to reduce greenhouse gas emissions and meet Net Zero targets (UN 2021). This is leading to exponential increases in renewable energy infrastructure and associated land‐use change (IEA 2021). Demand for land for other uses, including agriculture and nature recovery, is also intensifying, with global targets aiming to conserve or protect at least 30% of terrestrial and inland water areas by 2030 (UN 2022). Consequently, the COP28 Joint Statement on Climate, Nature and People emphasises that global climate change targets cannot be achieved without addressing climate change, biodiversity loss and land degradation in a synergetic manner (UN 2023a). Renewable energy sites, and in particular ground‐mounted solar photovoltaic (PV) farms, offer much potential to achieve this (Randle‐Boggis et al. 2020; Tölgyesi et al. 2023).

Solar PV accounted for three quarters of global renewable energy capacity additions in 2023 (IEA 2024), and utility scale PV, predominantly ground‐mounted solar farms, accounted for ~52% of total deployment (IEA 2023). Compared to conventional energy technologies, the power density (i.e., the land area needed to produce a given amount of power) of solar farms is relatively low (Capellán‐Pérez et al. 2017), which is a concern given that land‐use change presents an equivalent or greater threat to biodiversity than climate change (IPBES 2019). Moreover, anticipated future land‐use changes, driven by policy, developments in technology (Burgess and Morris 2009), changing demand for specific products (Angus et al. 2009) and climate change (plus mitigation attempts; Oliver and Morecroft 2014), enhance risks to biodiversity globally. Future land‐use scenarios, which consider varying socioeconomic and climatic factors, offer insight into the potential consequences of future land‐use changes, with projections indicating that differences in future land management and landscape composition will have significant implications for biodiversity (Brown et al. 2022; Newbold et al. 2015; Redhead et al. 2020; Pereira et al. 2024).

While land‐use change for solar farms presents risks to biodiversity (Hernandez et al. 2015; Rehbein et al. 2020), there are also opportunities to embed benefits for groups such as insect pollinators (Blaydes et al. 2021), with implications for a range of ecosystem services beneficial to human society and ecosystems (Potts et al. 2016; Walston et al. 2021). Using solar farms to support pollinator conservation is a relatively novel concept but could be achieved through a range of mechanisms, including providing microclimatic variation, increasing landscape heterogeneity and connectivity, and adapting site management practices (Blaydes et al. 2021). Indeed, evidence suggests that solar farms managed with a biodiversity focus could support a greater abundance and diversity of pollinators compared to similar land uses (Randle‐Boggis et al. 2020; Walston et al. 2021). Such action could mitigate declining population trends reported for some groups, including bumblebees (Ghisbain et al. 2023), which are critical pollinators in agricultural systems (Hutchinson et al. 2021; Kleijn et al. 2015). Managing solar farms to provide a continuous supply of bumblebee foraging resources (pollen and nectar from flowering plants) and nest sites (many species nest underground) could support or enhance populations and pollination services (Blaydes et al. 2022, 2024), potentially resulting in benefits to wider ecosystem conservation (Potts et al. 2016), increased agricultural yields (Walston et al. 2018, 2023) and lead to income streams from policy incentives and nature markets (UN 2023b). Moreover, given the typical lifespan of solar farms is 25–40 years (Solar Energy UK 2022), appropriately managed solar farms could ensure habitats are retained for decades, potentially moderating impacts of future habitat loss in the wider landscape (Brown et al. 2016).

Although understanding of pollinator response to solar farms in the present day is increasing, potential responses to these developments as wider landscapes undergo change remain uninvestigated. Consequently, the overall aim of this study was to determine whether solar farms currently in operation across the nation could support bumblebees in the future amid wider land‐use change occurring beyond site boundaries. To achieve this, we (i) predicted and compared bumblebee density in solar farms, surrounding foraging zones and wider landscapes between the present day and future scenarios and (ii) assessed which land‐use changes drove changes in bumblebee density.

## 2 Methods

**FIGURE 1:**
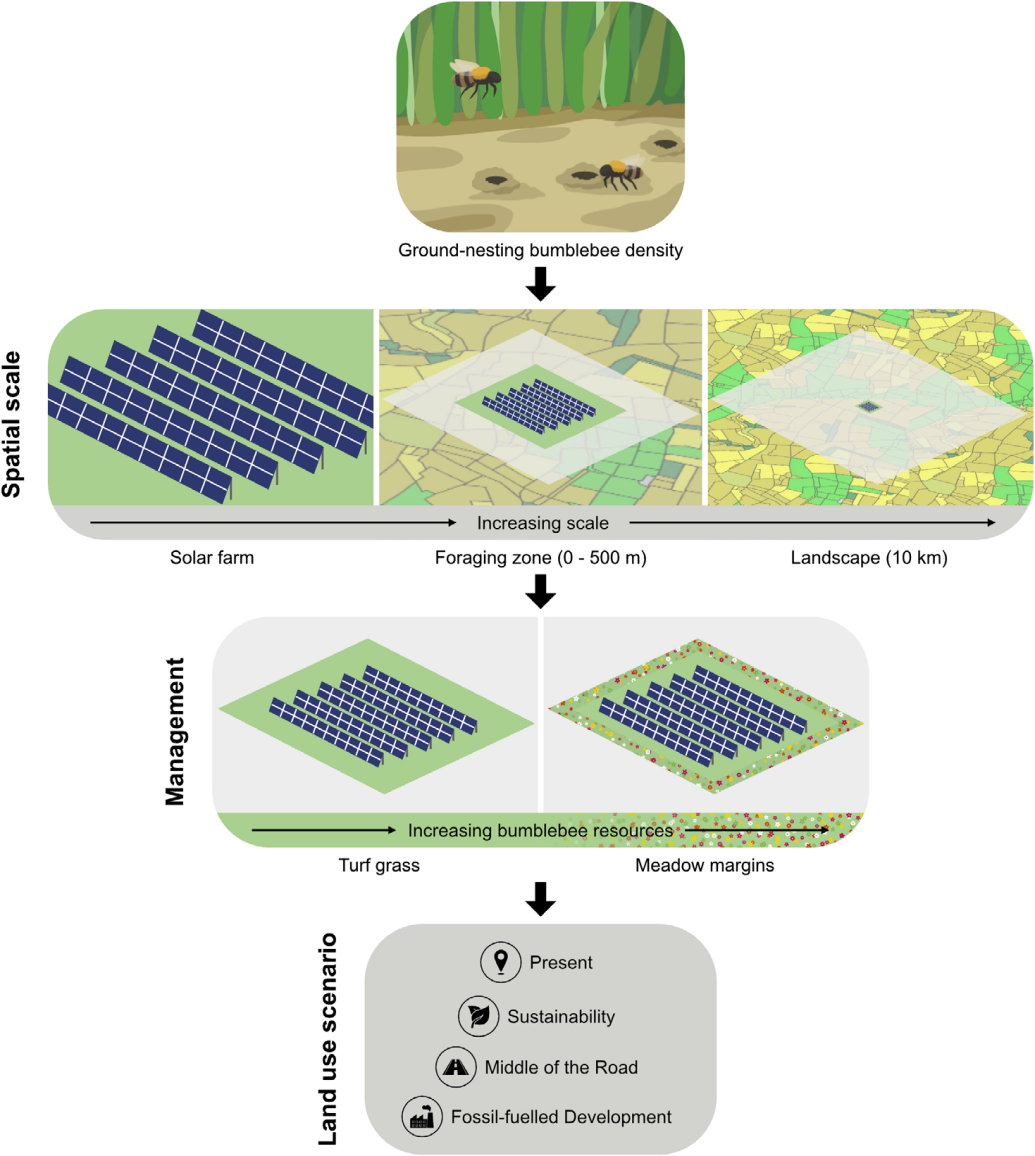
Schematic summary of the scenarios in which ground‐nesting bumblebee density (foraging workers and new queens) was predicted, in relation to spatial scale, solar farm management and land‐use scenario. In turf grass solar farms, vegetation across the whole site is equivalent to improved grassland (i.e., higher productivity grassland used for agriculture) whereas edges of meadow margin solar farms are equivalent to unimproved grassland (i.e., lower productivity semi‐natural grassland), which provide more bumblebee resources. Land‐use scenario icons were reproduced from the Noun Project (https://thenounproject.com).

To investigate bumblebee response to solar farms amid wider land‐use changes, we used a Geographic Information System (GIS) and a process‐based pollinator model to estimate the impacts of solar farm management strategies on bumblebee density in solar farms, surrounding foraging zones and landscapes (Figure 1). We explored the impacts of two management strategies that represent common industry practice in the present day and under three different socioeconomic futures for 2050, based on the established Representative Concentration Pathways and the Shared Socioeconomic Pathways (Brown et al. 2022; O'Neill et al. 2020; Figure 1). This involved four key steps: (i) solar farm digitisation and creation of foraging zones and landscapes, (ii) preparation of land‐use maps, (iii) pollinator modelling and (iv) statistical analysis. The approach is applied to Great Britain, given data and model availability, but could be replicated for other regions.

### 2.1 Solar Farm Digitisation and Creation of Foraging Zones and Landscapes

Operational, ground‐mounted solar farms in Great Britain (England, Scotland and Wales) were located using the Renewable Energy Planning Database quarterly extract for December 2021 (UK Government 2021a). Solar farms (*n* = 1042) were then digitised using aerial imagery in ArcGIS Pro (version 2.5.0; Esri 2023) or Google Earth Pro (version 7.3.3.7721; Google 2023). Solar farm boundaries (the fence line) and solar panels within were digitised by creating polygons. Margin areas within the solar farm boundary (areas not covered by solar panels) were generated by erasing solar panel polygons from solar farm boundary polygons. The size of solar farms ranged from 2559 to 1,241,573 m^2^, with a mean area of 139,403 ± 4020 m^2^. Solar farm shape was also variable, with some solar farms occupying a single land parcel and others spanning multiple fields. Consequently, there was variation in margin area and distribution within sites, but on average, margin areas occupied 28.9% ± 0.3% of total solar farm boundary area.

**FIGURE 2:**
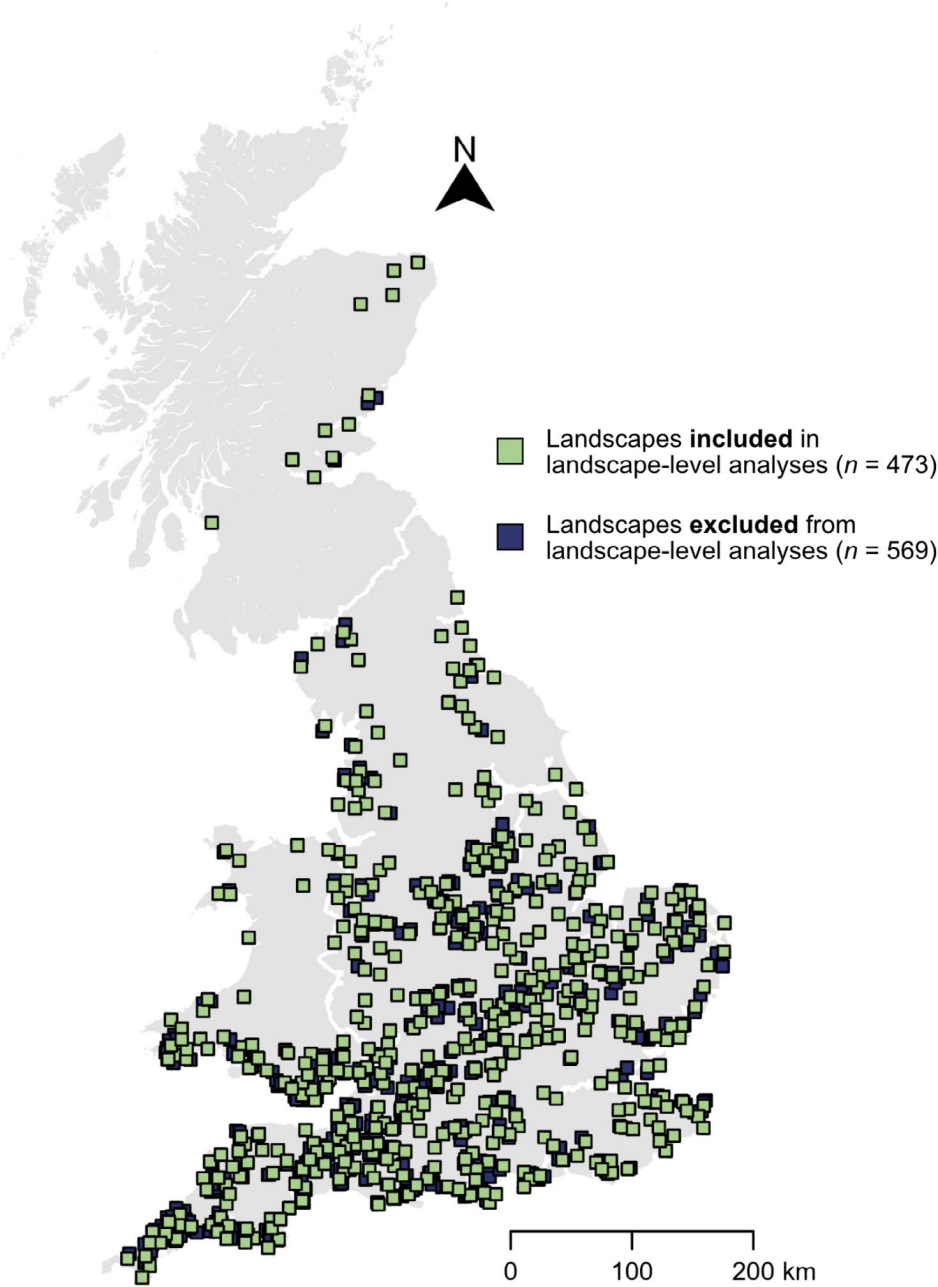
The locations of all solar farm landscape squares in Great Britain (England, Wales and Scotland; *n* = 1042), where green squares represent landscapes included in landscape‐level analyses (*n* = 473) and blue squares represent those excluded (*n* = 569) due to overlap (> 25%). Map lines delineate the study area and do not necessarily depict accepted national boundaries.

To represent wider landscapes surrounding each solar farm, a 10 km × 10 km landscape (see below for details of landcover data used for each scenario) centred on each solar farm was created (*n* = 1042), although landscape squares with significant overlap (> 25%) were excluded from landscape‐scale analyses (*n* = 569; Figure 2, Text S1; Gardner et al. 2021). Buffer zones extending 0–500 m from the solar farm boundaries were created to represent bumblebee foraging zones (*n* = 1042). Distances of 500 m were based on the average foraging distance of a bumblebee colony, although individual workers can travel further dependent on landscape quality (Blaydes et al. 2022; Redhead et al. 2015). Solar farms and foraging zones were rasterised at 10 × 10 m pixel resolution for input into the pollinator model.

### 2.2 Preparation of Land‐Use Maps

One present day and three future scenarios were used to explore the impact of land‐use change on bumblebee density inside solar farms, foraging zones and wider landscapes, represented by land use maps derived from the UK's Representative Concentration Pathways (RCPs) and Shared Socioeconomic Pathways (SSPs; Brown et al. 2022). UK‐RCP‐SSPs enable exploration of potential future land use in the UK as a result of future climate and socioeconomic conditions, and projections span from 2020 to 2080 in decadal time slices (Oliver and Morecroft 2014), but we focus on 2050 given the UK's Net Zero emissions targets (UK Government 2021b). Whilst there are five scenarios, we focus on *Sustainability* (RCP2.6‐SSP1; ‘SSP1’), *Middle of the Road* (RCP4.5‐SSP2; ‘SSP2’) and *Fossil‐fuelled Development* (RCP8.5‐SSP5; ‘SSP5’) to represent contrasting futures with different implications for climate and land‐use change (Table 1). The *Middle of the Road* projection for 2020 was used to represent a present‐day scenario. All of the UK‐RCP‐SSP land‐use maps can be obtained from online repositories (CRAFTY‐GB 2023), and a detailed narrative accompanies each UK‐SSP, describing land‐use changes and their drivers (Harrison et al. 2021a, 2021b, 2021c).

**TABLE 1:** Descriptions of UK‐RCP‐SSP scenarios including distinguishing socioeconomic features and main land use outcomes.

Scenario	Description	Distinguishing features	Main land use outcomes
*Sustainability* (RCP2.6‐SSP1)	A sustainable and cooperative society with a low‐carbon economy and high capacity to adapt to climate change	Novel forms of sustainable agriculture with strong societal support	Decreasing area of intensive agriculture, greater multifunctionality of agricultural land
		Low demand for livestock products, but preference for grass‐fed production	Move away from livestock production and decrease in pastoral area
		Preference for native tree species in forestry	Substanstial shift towards native species in forests, depending on suitability
*Middle of the Road* (RCP4.5‐SSP2)	A highly regulated society that continues to rely on fossil fuels, but with gradual increases in renewable energy, resulting in intermediate adaptation and mitigation challenges	Established forms of agriculture with potential for intensification	Intensification and increasing efficiency of agriculture, leading to intensive area declines
		Increasing demand for timber and forest‐based carbon sequestration	Large increase in forest area, dominated by non‐native tree species
		Low demand for grass‐fed livestock products	Large decrease in intensive pasture area, most livestock production feed‐based
*Fossil‐fuelled Development* (RCP8.5‐SSP5)	A technologically advanced world with a strong economy that is heavily reliant on fossil fuels, but with capacity to adapt to the impacts of climate change	Increasing demands for urban areas and food production	High pressure on land area and strong competition between land uses
		Increasing intensification options	Very high levels of agricultural intensification, supporting large increases in production
		Removal of protected areas and low demands for related ecosystem services	Expansion of productive land uses into natural areas, with abandonment in upland and marginal areas

**FIGURE 3:**
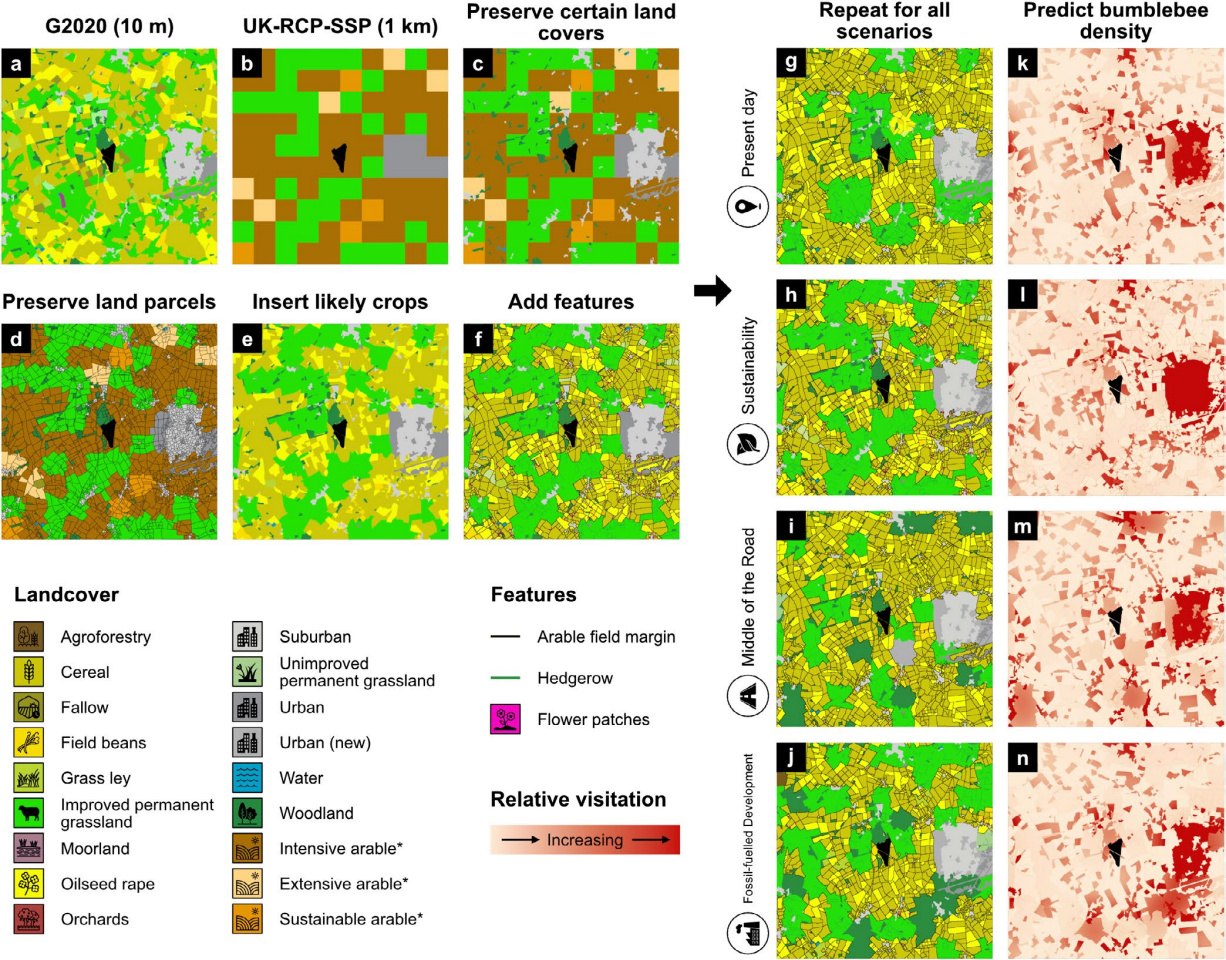
A summary of the downscaling process required to prepare land use maps for use with the pollinator model (left) and example model inputs and outputs (right). The process follows an example 10 km × 10 km landscape surrounding a solar farm where (a) shows the present day G2020 landcover map, (b) shows the future UK‐RCP‐SSP land use map (*Sustainability* shown here), (c) shows the result of a raster overlay using conditional statements to preserve certain landcovers, (d) shows the result of calculating the majority landcover class within each land parcel, (e) demonstrates the insertion of likely crops into arable land parcels and (f) presents the final hybrid landcover map, with added features (arable field margins, hedgerows and flower patches). Landcover classes with an asterisk are those that were assigned specific crop types as part of the downscaling process. This process was repeated for each land use scenario and (g–j) show example hybrid landcover maps inputted into the pollinator model, where (k–n) show model outputs of predicted relative bumblebee visitation. Displayed outputs correspond to simulations with the solar farm at the centre managed as turf grass. Land use scenario and landcover icons were reproduced from the Noun Project (https://thenounproject.com).

UK‐RCP‐SSP land‐use maps were downscaled due to their coarse spatial resolution compared with the requirements of the pollinator model (Figure 3). The model requires high spatial resolution landcover information to simulate foraging processes and uses a 10 m landcover map typically derived from the UKCEH Landcover Map 2015 (Rowland et al. 2017), with added Ordnance Survey orchard polygons and 2016 crop location information derived from rural payments agency databases (hereafter the ‘G2020 map’; Figure 3a; Gardner et al. 2020). In comparison, UK‐RCP‐SSP maps provide information about the dominant land use at 1 km resolution (Figure 3b). Portions of other landcover types are likely to exist within these 1 km pixels and therefore, to capture land‐use information from UK‐RCP‐SSP maps, while retaining the spatial detail of the G2020 map, a hybrid landcover map was created for each UK‐RCP‐SSP scenario.

Additionally, the G2020 map consists of 24 landcover classes that the pollinator model is parameterised for (i.e., those that have been scored by pollinator experts in terms of floral cover, floral attractiveness and nesting attractiveness), whereas the UK‐RCP‐SSP maps consist of 17 broader land‐use classes that must likewise be translated. Each UK‐RCP‐SSP land‐use class was therefore assigned an equivalent landcover class from the G2020 map. Where there was not a direct equivalent in G2020, land‐use classes were assigned scores made up of different proportions of relevant existing G2020 landcover scores (for further details see Text S1 and Table S1).

Land‐use transition decisions were implemented in ArcGIS Pro (Esri 2023). Each UK‐RCP‐SSP land‐use map was resampled from 1 km to 10 m resolution to ensure every cell had a direct equivalent in the G2020 map. Next, a raster overlay using conditional statements was undertaken, whereby the location and attribution of the G2020 landcover map and UK‐RCP‐SSP land‐use maps informed the creation of hybrid rasters such that the conversion of pixels to new coarse‐scale landcovers was dependent on their current fine‐scale landcover (Figure 3c). To preserve a realistic landscape structure, land parcels from the present day were reintroduced and within each parcel, the majority landcover class was calculated (Figure 3d). Following this, broad arable land‐use types (i.e., intensive arable, extensive arable and sustainable arable) were assigned specific crop types (cereal, oilseed rape, field beans or grass ley) based on arable land‐use descriptions (Redhead et al. 2020) and common crop rotations identified across the UK (Upcott et al. 2023; Figure 3e). Appropriate agri‐environment features such as flower patches, field margins and hedgerows were then added to represent further agroecological differences between scenarios consistent with interpretations of the UK‐SSP narratives (Figure 3f). For example, field margins were wider and total hedgerow length was greater in the *Sustainability* scenario compared to other scenarios. Agri‐environment features were generated in R (version 4.2.3; R Core Team 2023) and for more information, see Texts S3 and S4.

Once land‐use maps had been prepared, the mean percentage cover of each landcover class and feature was calculated inside 0–500 m foraging zones (*n* = 1042) and 10 km landscapes (*n* = 473) surrounding each solar farm across each scenario. The change in area of each landcover class and feature was also calculated between the present day and each future scenario in foraging zones (*n* = 1042) and landscapes (*n* = 473). All landscapes were rasterised at 10 × 10 m pixel resolution for input into the pollinator model.

### 2.3 Pollinator Modelling

poll4pop is a process‐based model derived from the Lonsdorf model (Lonsdorf et al. 2009), developed by Olsson et al. (2015) and Häussler et al. (2017). The model has been parameterised and validated for the UK and simulates the foraging and population processes of bees to predict their spatially explicit abundance in a given landscape using input landcover information, foraging and nesting habitat preferences, population density and movement range estimates (Gardner et al. 2020). In this study, we simulate ground‐nesting bumblebees, given that bees are the most significant crop pollinators (Hutchinson et al. 2021), bumblebees are generally the most mobile bees, and ground nesting is the most common bumblebee guild (Falk 2015). Model outputs consist of spatially explicit predictions of the abundance of foraging bumblebee workers, nests and new queens. However, we focus on foraging worker abundance as this signals forage and nest site availability but include the abundance of new queens in some analyses.

To predict bumblebee abundance, the model requires a high‐resolution rasterised landcover map for each landscape, where each landcover class is accompanied by parameter values representing (i) the floral cover it provides during each season, (ii) the attractiveness of its floral resources (where attractiveness reflects the nutritional quality of the resource) and (iii) the attractiveness of the landcover class in terms of nesting opportunities. Parameter values are expert‐derived, and attractiveness scores are specific to ground‐nesting bumblebees (Gardner et al. 2020).

A resource mapping function uses these parameters to convert the input rasterised landscape into separate maps that represent the distribution of foraging resources (seasonally resolved) and nesting resources. The model then seeds nests in the landscape according to the distribution of nesting resources, and a foraging function distributes foragers from the nests across foraging resources, assuming foraging bumblebees spend more time in proximate and better‐quality foraging areas. Next, a growth function relates the number of bumblebees produced per nest to the amount of foraging resources gathered, enabling the amount and accessibility of foraging resources to influence the population size. New reproductive females (i.e., new queens) produced by each nest are dispersed across the landscape, and the availability of nesting resources limits the number that survive to found their own nests the following year. A foraging distance of 530 m and a dispersal distance to new nest sites of 1000 m were used, based on values derived from the literature (Gardner et al. 2020). For more information about the model, see Gardner et al. (2020) and Gardner et al. (2024), and for further details about the inputs and parameters used in this study, see Text S5 and Tables S2–S4.

poll4pop was run for solar farm landscapes using the hybrid present day, *Sustainability*, *Middle of the Road* and *Fossil‐fuelled Development* land‐use scenario maps. For all scenarios, two solar farm management strategies, providing different levels of floral and nesting resources to bumblebees, were applied. Firstly, the improved grassland landcover class (i.e., higher productivity grassland used for agriculture) represented solar farms managed as turf grass, offering some bumblebee resources (*turf grass*). Secondly, improved grassland was applied in combination with the unimproved meadow landcover class (i.e., lower productivity, semi‐natural grassland offering high levels of resources to bumblebees) to create a management strategy whereby areas within blocks of solar panels were turf grass, but margins provided more resources (*meadow margins*, Tables S2 and S3). This meant each solar farm underwent a total of eight simulations (four land‐use scenarios multiplied by two solar farm management strategies), and output data are available from the Dryad Digital Repository (Blaydes, Gardner, et al. 2025).

For each simulation, mean foraging bumblebee density (per 100 m^2^) and mean new bumblebee queen density (per 100 m^2^) were calculated within solar farms (*n* = 1042), 0–500 m foraging zones (*n* = 1042), and 10 km landscapes (*n* = 473) in each scenario by dividing predicted bumblebee abundance values by area. Bumblebee density was used in analyses to normalise for the effect of area given different solar farm and foraging zone sizes. The total foraging and nesting resources available in each solar farm foraging zone (*n* = 1042) and landscape (*n* = 473) were also calculated across three seasons (early spring, late spring and summer).

To investigate differences between the present day and future, the change in foraging bumblebee density within each solar farm (*n* = 1042), foraging zone (*n* = 1042), and landscape (*n* = 473) was calculated between the present day and each future scenario.

### 2.4 Statistical Analysis

All statistical analyses were undertaken in R (R Core Team 2023) and to quantify differences in bumblebee density across scenarios, the differences in both mean foraging bumblebee density and mean new bumblebee queen density were tested using analysis of variance (ANOVA), followed by post hoc Tukey tests. To meet test assumptions, foraging bumblebee density data were transformed via Box–Cox methods to ensure normality, but this was not necessary for new bumblebee queen density data given their already normal distribution. Analyses were performed separately for solar farms managed as turf grass and those with meadow margins within each land‐use scenario, given that only one solar farm management scenario was tested at the landscape scale. ANOVA and post hoc Tukey analyses were also performed to quantify the differences in foraging and nesting resources in solar farm foraging zones and landscapes.

To assess the drivers of change from present day to future scenarios, changes in landcover class and feature area were entered as variables into generalised linear models (GLMs) to assess which changes had a significant impact on changes in foraging bumblebee density. Changes in new bumblebee queen density were not explored given the similarity to foraging bumblebee density results in ANOVA analyses.

Nine key landcover classes (agroforestry, cereal, grass ley, field beans, improved permanent grassland, oilseed rape, unimproved permanent grassland, urban and woodland) were included as continuous, explanatory variables in GLMs, selected as they either made‐up large areas of landscapes surrounding solar farms or because of their value to bumblebees. The woodland landcover class included in GLMs represented multiple woodland classes grouped together, which varied in type (broadleaf, coniferous or mixed), function (productive, conservation or multifunctional) and whether they were native or non‐native (see Text S2 and Table S1 for more details). In total, 12 GLMs were built to investigate the drivers of foraging bumblebee density change in (i) solar farms managed as turf grass, (ii) solar farms managed with meadow margins, (iii) foraging zones surrounding solar farms managed as turf grass and (iii) landscapes containing a solar farm managed as turf grass, each with three models representing change from the present day to each future scenario. Assumptions of normality and equal variances were visually checked using histograms and Q–Q plots.

## 3 Results

### 3.1 Bumblebee Response to Land‐Use and Management Scenarios

**FIGURE 4:**
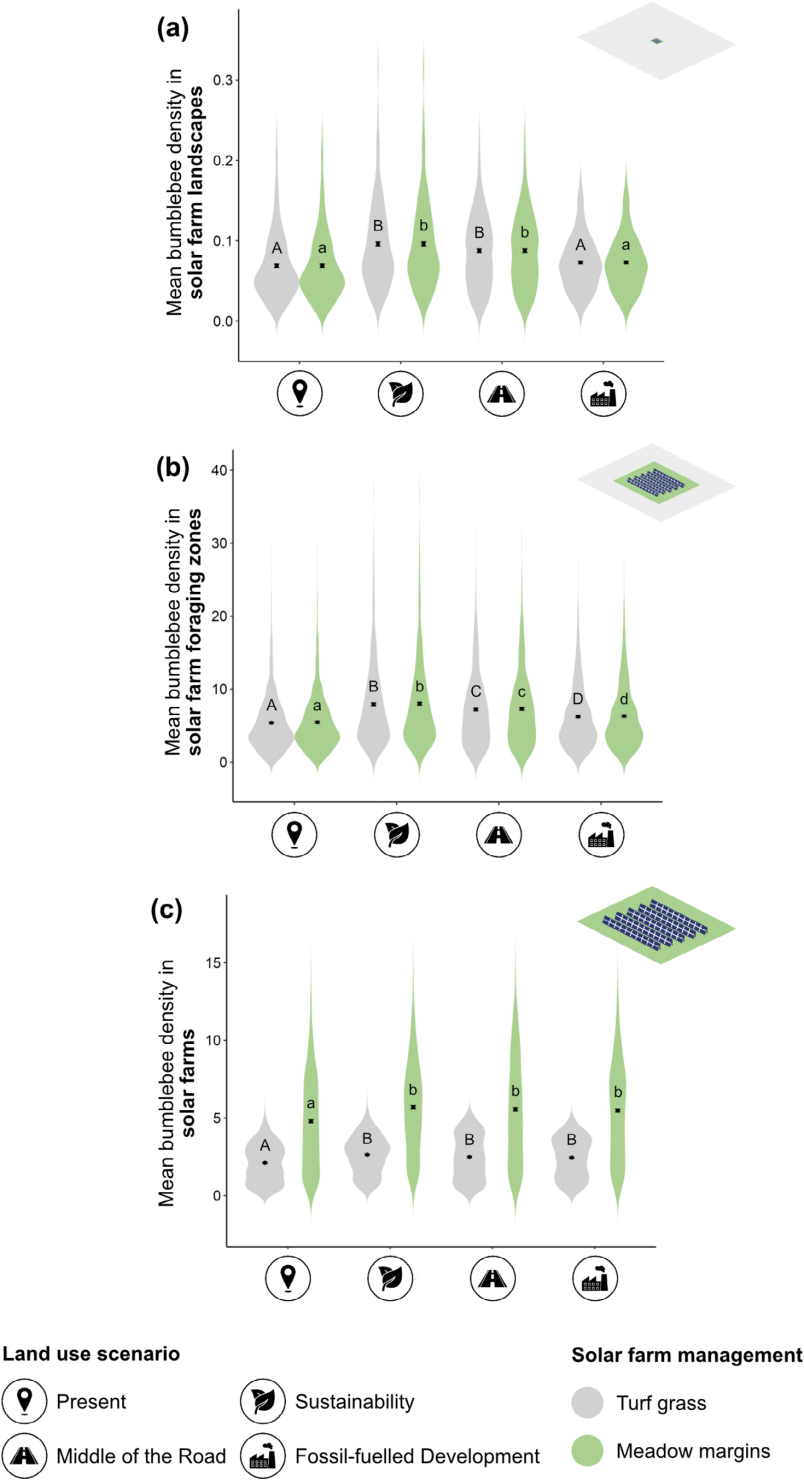
Distributions of spatially‐averaged mean foraging bumblebee density (per 100 m^2^) in (a) 10 km landscapes surrounding solar farms (*n* = 473), (b) 0–500 m foraging zones surrounding solar farms (*n* = 1042) and (c) solar farms (*n* = 1042) across land use scenarios. Black points show the sample‐level mean and error bars represent the standard error on this sample‐level mean. Within each plot, points that share letters are not significantly different at the *p* < 0.05 level according to ANOVA and Tukey post hoc analyses. Upper‐case letters present the results of ANOVA and Tukey analyses relating to solar farms managed as turf grass (grey) and lower‐case letters present results relating to solar farms managed with meadow margins (green). Data were transformed before analysis using Box–Cox methods to meet statistical test assumptions. Land use scenario icons were reproduced from the Noun Project (https://thenounproject.com).

At the landscape scale (from the solar farm boundary to 10 km away), solar farm management was inconsequential, and foraging bumblebee density was greater under *Sustainability* and *Middle of the Road* scenarios, compared with the present day and *Fossil‐fuelled Development* (Figure 4a, Table S5). Similarly, at the foraging zone scale (from the solar farm boundary to 500 m away), solar farm management had no effect, and bumblebee density was greatest under *Sustainability*, followed by *Middle of the Road*, *Fossil‐fuelled Development* and lowest in the present day (Figure 4b, Table S6). At the solar farm scale (within the solar farm boundary), bumblebee density was higher in solar farms under future scenarios, compared with the present day (Figure 4c, Table S7). In contrast, management was the strongest driver of differences in bumblebee density within solar farms, and density was always higher in solar farms managed with meadow margins (i.e., those with inter‐panel vegetation equivalent to improved grassland but with margins equivalent to unimproved grassland), compared to turf grass (i.e., those where both interpanel vegetation and margin areas are equivalent to improved grassland), regardless of land‐use scenario (Figure 4c, Table S7). Mean increases in bumblebee densities inside meadow margin solar farms were greatest in the present day (126%), followed by *Fossil‐fuelled Development* (124%), *Middle of the Road* (123%) and lowest in *Sustainability* (117%; Figure 4c). The results were similar for new bumblebee queen density across spatial scales (Text S6, Figure S1, Tables S8–S10) and the availability of bumblebee foraging and nesting resources in each land‐use scenario broadly mirrored patterns seen in mean bumblebee densities, with the greatest amounts present in *Sustainability* and *Middle of the Road* (Text S7, Figure S2, Tables S11–S14).

### 3.2 Drivers of Change in Bumblebee Density

**FIGURE 5:**
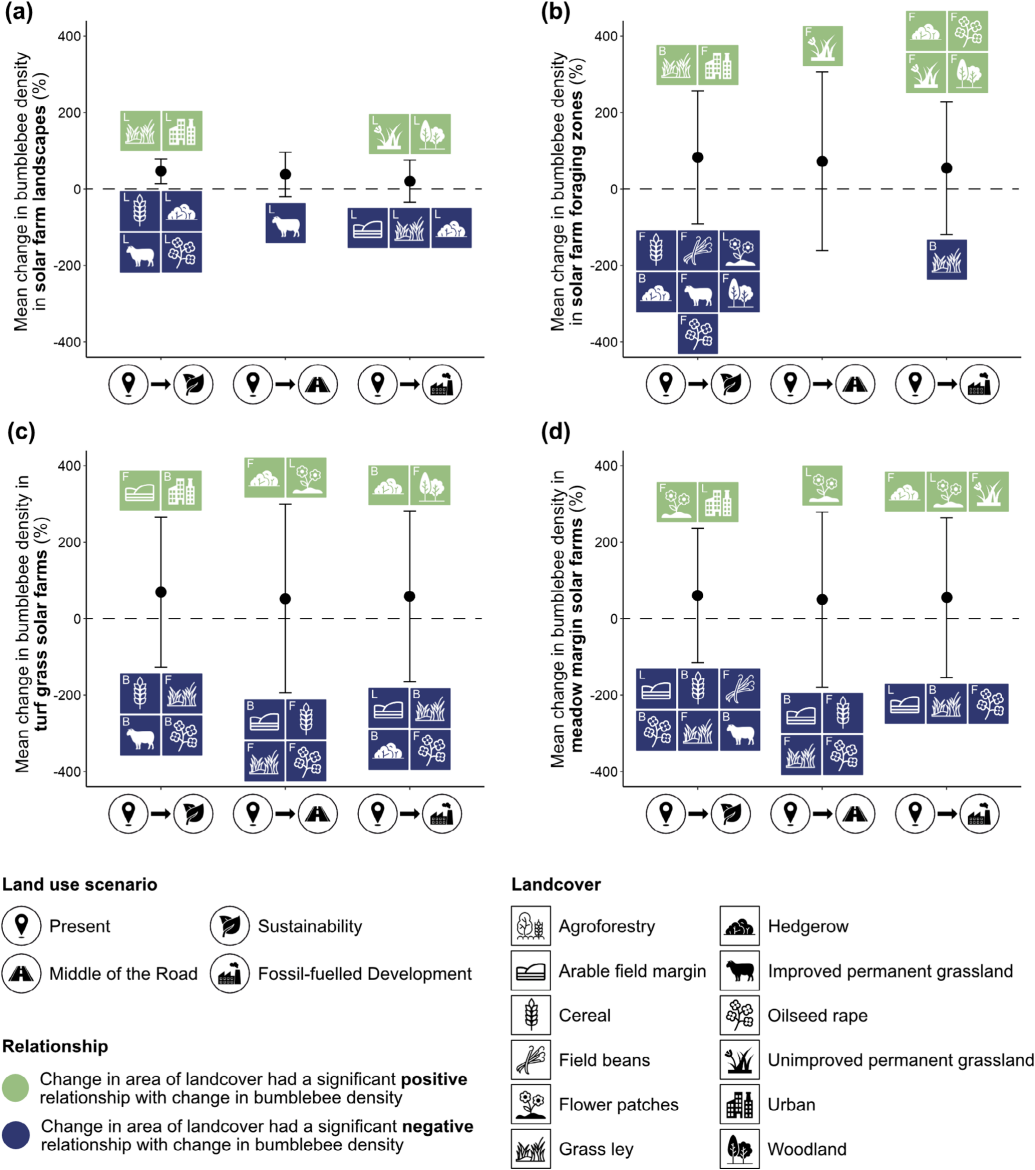
The overall mean change (± standard deviation) in foraging bumblebee density from the present day to each future land use scenario at the (a) solar farm landscape (*n* = 473), (b) solar farm foraging zone (*n* = 1042) and (c, d) solar farm scale (*n* = 1042). Coloured icons summarise the relationships between the change in foraging bumblebee density and the change in the area of key landcover classes based on results from 12 generalised linear models. Green icons indicate the landcover had a significant positive relationship with change in bumblebee density and blue icons indicate a significant negative relationship. Significant landcover changes may have occurred at the foraging zone scale (F), landscape scale (L), or both (B), indicated by the letter in the top left hand corner of each icon. Land use scenario and landcover icons were reproduced from the Noun Project (https://thenounproject.com).

**FIGURE 6:**
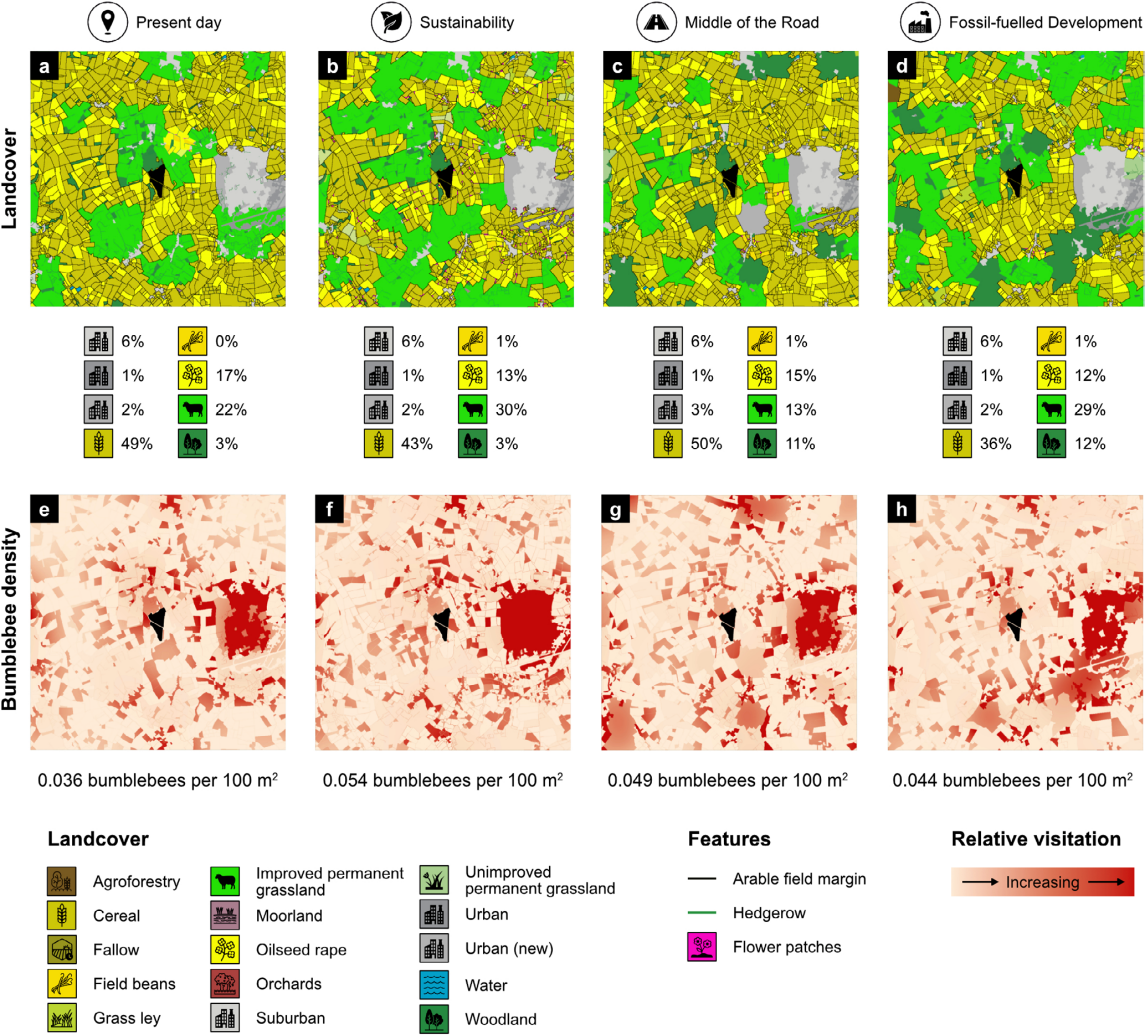
An example 10 km × 10 km landscape surrounding a solar farm, where (a—d) show landcover composition and (e—h) show bumblebee density across this landscape under the present day, *Sustainability*, *Middle of the Road*, and *Fossil‐fuelled Development* scenarios. Percentages beneath (a—d) indicate the percentage cover of each of the eight key landcovers present in this landscape and values under (e—h) represent bumblebee density. Displayed outputs correspond to simulations with the solar farm at the centre managed as turf grass. Land use scenario and landcover icons were reproduced from the Noun Project (https://thenounproject.com).

Mean foraging bumblebee density in solar farms, foraging zones and landscapes increased between the present day and all three future land use scenarios (Figures 5 and 6). Changes in bumblebee density varied by land‐use scenario, solar farm management strategy and spatial scale, driven by changes in the area of certain landcover classes, including crops such as cereal, but also improved grassland, woodland and urban landcovers (Figure 5, Table 2, Tables S15–S18, Figure S3).

**TABLE 2:** The mean percentage cover (± standard error) of landcover classes and agri‐environment features surrounding solar farms in 0–500 m foraging zones (*n* = 1042) and 10 km landscapes (*n* = 473) across land use scenarios.

Landcover/feature	Land use scenario							
	Present		*Sustainability*		*Middle of the Road*		*Fossil‐fuelled Development*	
	0–500 m	10 km	0–500 m	10 km	0–500 m	10 km	0–500 m	10 km
Improved permanent grassland	36 ± 1	32 ± 1	38 ± 1	28 ± 1	21.9 ± 0.8	16.7 ± 0.6	34 ± 1	28 ± 1
Cereal	32.9 ± 0.9	29 ± 1	28.0 ± 0.8	28 ± 1	31.9 ± 0.8	30 ± 1	22.0 ± 0.8	22.0 ± 0.9
Woodland	8.9 ± 0.4	9.6 ± 0.4	10.5 ± 0.5	12.0 ± 0.5	21.2 ± 0.7	21.2 ± 0.7	17.6 ± 0.6	16.8 ± 0.5
Oilseed rape	10.8 ± 0.4	9.3 ± 0.3	9.3 ± 0.3	9.3 ± 0.3	10.1 ± 0.3	9.6 ± 0.3	7.5 ± 0.3	7.1 ± 0.3
Urban	3.8 ± 0.3	4.2 ± 0.2	5.4 ± 0.3	5.1 ± 0.3	5.3 ± 0.3	5.2 ± 0.3	7.0 ± 0.4	7.7 ± 0.3
Suburban	3.9 ± 0.2	8.3 ± 0.3	3.9 ± 0.2	8.3 ± 0.3	3.9 ± 0.2	8.3 ± 0.3	3.9 ± 0.2	8.3 ± 0.3
Water	1.4 ± 0.2	4.2 ± 0.4	1.4 ± 0.2	4.2 ± 0.4	1.4 ± 0.2	4.2 ± 0.4	1.4 ± 0.2	4.2 ± 0.4
Arable field margin	0.62 ± 0.02	0.0051 ± 0.0002	2.21 ± 0.06	0.0215 ± 0.0007	0.73 ± 0.02	0.0066 ± 0.0002	0.67 ± 0.03	0.0063 ± 0.0002
Hedgerow	0.75 ± 0.01	0.0061 ± 0.0001	1.63 ± 0.03	0.0136 ± 0.0002	0.71 ± 0.01	0.0057 ± 0.0001	0.59 ± 0.01	0.00503 ± 0.00009
Agroforestry	0.0001 ± 0.0001	0.0008 ± 0.0005	0.2 ± 0.1	0.2 ± 0.0	0.6 ± 0.1	0.6 ± 0.0	2.1 ± 0.2	1.9 ± 0.1
Moorland	0.6 ± 0.1	0.6 ± 0.1	0.6 ± 0.1	0.6 ± 0.1	0.6 ± 0.1	0.6 ± 0.1	0.6 ± 0.1	0.6 ± 0.1
Unimproved permanent grassland	0.4 ± 0.1	1.0 ± 0.1	0.2 ± 0.1	0.5 ± 0.1	0.8 ± 0.2	0.7 ± 0.1	0.8 ± 0.2	0.5 ± 0.1
Field beans	0.0 ± 0.0	0.1 ± 0.1	0.7 ± 0.1	0.7 ± 0.1	0.5 ± 0.1	0.4 ± 0.0	0.9 ± 0.1	0.87 ± 0.04
Very extensive pasture	0.19 ± 0.05	0.12 ± 0.02	0.21 ± 0.06	0.27 ± 0.05	0.8 ± 0.1	0.9 ± 0.1	0.7 ± 0.1	0.5 ± 0.1
Beaches, sand dunes or planes	0.3 ± 0.1	0.7 ± 0.1	0.3 ± 0.1	0.7 ± 0.1	0.3 ± 0.1	0.7 ± 0.1	0.3 ± 0.1	0.7 ± 0.1
Grass ley	0.02 ± 0.01	0.025 ± 0.006	0.46 ± 0.07	0.74 ± 0.04	0.26 ± 0.05	0.27 ± 0.02	0.45 ± 0.07	0.42 ± 0.02
Scrub	0.0 ± 0.0	0.0 ± 0.0	0.9 ± 0.2	0.7 ± 0.1	0.0006 ± 0.0006	0.011 ± 0.003	0.0 ± 0.0	0.009 ± 0.003
Salt marsh	0.2 ± 0.1	0.5 ± 0.1	0.2 ± 0.1	0.5 ± 0.1	0.2 ± 0.1	0.5 ± 0.1	0.2 ± 0.1	0.5 ± 0.1
Flower patches	0.006 ± 0.002	0.000060 ± 0.000005	0.64 ± 0.02	0.0066 ± 0.0002	0.032 ± 0.005	0.00029 ± 0.00002	0.067 ± 0.007	0.00066 ± 0.00003
Wetland	0.11 ± 0.03	0.16 ± 0.03	0.11 ± 0.03	0.16 ± 0.03	0.11 ± 0.03	0.16 ± 0.03	0.11 ± 0.03	0.16 ± 0.03
Orchards	0.0 ± 0.0	0.0 ± 0.0	0.0 ± 0.0	0.00012 ± 0.00006	0.0 ± 0.0	0.003 ± 0.0002	0.000 ± 0.002	0.0076 ± 0.0002
Unimproved meadow	0.0 ± 0.0	0.0 ± 0.0	0.0 ± 0.0	0.0002 ± 0.0002	0.0 ± 0.0	0.00010 ± 0.00006	0.0 ± 0.0	0.0003 ± 0.0003

*Note:* Features were overlaid onto landcover maps during pollinator modelling and therefore mean percentage cover values may total more than 100%.

At the landscape scale, foraging bumblebee density increased with increases in urban area and grass ley in *Sustainability*, increased with increases in unimproved permanent grassland and woodland area in *Fossil‐fuelled Development* and was driven by decreases in agricultural land and associated features, such as arable field margins, in all future scenarios (Figure 5a, Table 2, Figure S3).

Similarly, at the foraging zone scale, increases in foraging bumblebee density were driven by urban areas and grass ley in *Sustainability* (Figure 5b, Table 2, Figure S3). In contrast, changes in bumblebee densities were associated with changes in the area of semi‐natural habitats and oilseed rape in *Fossil‐fuelled Development*, and in addition, with unimproved permanent grassland in *Middle of the Road* (Figure 5b, Table 2, Figure S3). Moreover, changes in bumblebee density in the foraging zone showed a negative relationship with changes in the area of improved grassland, arable crops and semi‐natural habitats in *Sustainability* and with grass ley in *Fossil‐fuelled Development* (Figure 5b, Table 2, Figure S3).

As similar to the landscape and foraging zone scales, in *Sustainability*, an increase in urban area contributed to increases in foraging bumblebee density inside solar farms under both management strategies (Figure 5c,d, Table 2, Figure S3). In *Middle of the Road*, solar farm bumblebee density increased with flower patch area regardless of solar farm management, and with hedgerow area for solar farms managed as turf grass (Figure 5c,d, Figure S3). In *Fossil‐fuelled Development*, solar farm bumblebee density was driven by changes in semi‐natural habitats such as hedgerows, woodland and flower patches (Figure 5c,d, Table 2, Figure S3). Across all future scenarios, bumblebee density at the solar farm scale increased with decreases in the area of agricultural landcovers and their associated features (Figure 5c,d, Table 2, Figure S3).

## 4 Discussion

Solar farm management and surrounding land use, as dictated by land‐use scenario and interpreted in the downscaling, both had significant impacts on predicted bumblebee densities. Land‐use scenario had a greater impact on landscape‐scale and foraging zone bumblebee densities, with all future scenarios increasing densities compared with the present day. Although this may seem counterintuitive, present‐day landscapes surrounding solar farms are dominated by agriculture and contain high proportions of landcovers such as improved grassland and cereal, which provide few nesting and floral resources for bumblebees. Such landscapes could therefore become more suitable for bumblebees under future land‐use changes if other landcover types, which offer more bumblebee resources, are introduced. For example, in *Sustainability*, agricultural land area decreases due to reductions in food waste, reduced meat consumption and sustainable intensification practices in agroecosystems, and biodiversity is embedded into the management of remaining farmland (Harrison et al. 2021a). Similarly, in *Middle of the Road*, area of intensive agriculture declines and sustainable agriculture is promoted (Harrison et al. 2021b), while in *Fossil‐fuelled Development*, agricultural land is replaced by other land uses such as urban, all with implications for bumblebees and their resources (Harrison et al. 2021c). While land‐use changes across scenarios could lead to increased bumblebee densities in solar farm landscapes, it is important to note that these represent only a subset of landscapes across Great Britain. If all landscapes were considered (i.e., including those less dominated by agriculture), we would likely see different impacts of land‐use change on bumblebee resources and densities.

Although bumblebee densities generally increased between the present day and future, the highest bumblebee densities were associated with more environmentally sustainable futures. In *Sustainability*, urbanisation in solar farm landscapes was a key driver of bumblebee density change as its urban landcover was assumed to have relatively high floral and nesting resources for bumblebees, representing green cities and populations with greater environmental awareness (Harrison et al. 2021a). In *Middle of the Road*, shifts towards sustainable farming supported bumblebees at the solar farm scale, but an influx of floral resources in the landscape (from landcovers such as oilseed rape) attracted foraging workers away from solar farms. In *Fossil‐fuelled Development*, semi‐natural habitats were important drivers of bumblebee density at all scales, which may be because landscapes in this scenario are less hospitable for bumblebees (i.e., it is assumed there are no flowering noncrop species amongst agricultural landcovers, and new urban areas provide no floral resources) and the few remnants of remaining semi‐natural habitats are therefore highly valuable. Although both positive and negative bumblebee density changes could be attributed to changes in certain land uses, it is likely that these net impacts conceal underlying opposing impacts and further research to disentangle this interplay could be undertaken to increase understanding of bumblebee response to land‐use change.

Whilst differences were apparent between land‐use scenarios, they were limited and may have been underestimated for three principal reasons. Firstly, the future land‐use scenario storylines provided few details on the differences between arable land uses (Harrison et al. 2021a, 2021b, 2021c). Instead, descriptions of arable land‐use types and common crop rotations from the literature were used to define differences between the scenarios (Redhead et al. 2020; Upcott et al. 2023), but likely underestimated the variation in the crops grown between scenarios (Rial‐Lovera et al. 2017) and did not account for more innovative or diverse crop rotations, ultimately affecting bumblebee density predictions (Hass et al. 2019; Marja et al. 2018). This is particularly important given solar farm landscapes were dominated by agricultural land uses. Secondly, differences in land management approaches between scenarios may also have been underestimated as these were characterised by present‐day options, omitting consequences of new policies or practices which may be in place by 2050. In scenarios such as *Sustainability*, this may translate to a greater number or diversity of agri‐environment interventions (only grass margins, hedgerows and flower patches were represented here), leading to bumblebee gains, whereas in *Fossil‐fuelled Development*, intensive management may be common practice, with agrochemical application prevalent in attempts to maximise productivity (Harrison et al. 2021c). Pesticide effects are not included in the pollinator model and may reduce bumblebee abundance, leading to greater differences in bumblebee density predictions between some scenarios (Feltham et al. 2014; Whitehorn et al. 2012). Moreover, changes in bumblebee densities may have feedbacks on land management decisions (Synes et al. 2018) which are also likely to differ across the scenarios but were not possible to capture in this study. Lastly, whilst the original land‐use scenarios accounted for climate changes when determining landcover change (Brown et al. 2022), direct impacts of climate change on bumblebee density were not accounted for. We simulate guild‐level, rather than species‐level bumblebee density, giving the results some robustness to climate‐induced range shifts or species turnover. However, the pollinator model does not consider the direct impacts of weather on bumblebee density, which could lead to larger differences between future land‐use scenarios given impacts on bumblebee physiology (Soroye et al. 2020), phenology (Wyver, Potts, Edwards, Edwards, Roberts, and Senapathi 2023) and distribution (Wyver, Potts, Edwards, Edwards, and Senapathi 2023), and the extent to which scenario land‐use mitigates or exacerbates this. Further work is now needed to build on our initial, likely conservative results, to explore whether differences between scenarios widen if more radical agroecological approaches and climate change impacts can be defined, parameterised and simulated. Future studies could also test how bumblebee densities change in response to different crop compositions and agricultural intensity levels within scenarios through running sensitivity analyses, but this would only be feasible at smaller spatial scales given computational demand.

Although surrounding land‐use impacts dominated at the foraging zone and landscape scale, solar farm management was the strongest driver of bumblebee density at the solar farm scale. This was the case in solar farms across all land‐use scenarios, indicating that well‐managed sites could support both foraging bumblebees and new bumblebee queens in the future across a range of surrounding land‐use changes and levels of environmental sustainability. Solar farms can be managed to support and enhance insect pollinators through the provision of suitable habitat (Blaydes et al. 2021), and given their relatively long life spans, habitats within solar farms could be capable of supporting localised bumblebee populations amid wider land‐use changes. Our results suggest that solar farms could contribute to supporting bumblebees in the future when managed to enhance floral resources, given bumblebee density was greater inside solar farms managed with floral‐rich margins, compared to those managed as turf grass, supporting findings from other modelling (Blaydes et al. 2022) and field‐based studies (Blaydes et al. 2024), as well as industry assessments (Montag et al. 2016; Solar Energy UK 2025). Managing solar farms entirely as wildflower meadows would likely further increase bumblebee gains (Blaydes et al. 2022), and while this is thought to be relatively rare in reality, managing space between solar panel rows is possible (Tölgyesi et al. 2023; Meyer et al. 2023). However, this may become more common practice, especially in more environmentally sustainable scenarios. For example, in *Sustainability*, development focuses on minimising environmental impacts, and multifunctional land uses, including solar farms, are promoted (Harrison et al. 2021a).

In contrast, solar farm management had little impact on bumblebee densities at the foraging zone scale. Elevated bumblebee densities surrounding solar farms managed with meadow margins may have been expected, where the flower‐rich habitats within these sites could provide resources to support bumblebees outside of the solar farm, but no significant effect was found. Such effects have been detected in field studies, where enhanced bee visitation to soybean flowers was observed adjacent to solar farms (Walston et al. 2023). In this case, a greater area, or different distribution, of resource‐rich habitat may have been required inside solar farms to have beneficial impacts beyond the site boundary and further work could be undertaken to increase understanding, given the potential to increase bumblebee densities and local crop pollination services (Walston et al. 2018, 2023). Further research could explore how spatial arrangements of habitats within solar farms could be optimised to enhance the spillover of pollination services from wild pollinators, as well as the impacts of colocating solar farms and pollinator‐dependent crops (Armstrong et al. 2021).

There was also no effect of solar farm management on landscape‐scale bumblebee densities, but this was expected given the relative size of most solar farms compared with the landscape scale used in the study. Solar farms were also considered in isolation, where bumblebee density was modelled in each solar farm and its surrounding landscape individually, and overlapping landscapes were removed from landscape‐scale analyses. It is therefore unlikely that a single solar farm in the centre of a 10 km × 10 km landscape would impact bumblebee density at this scale. However, if the cumulative impacts of multiple well‐managed solar farms (i.e., providing greater areas of bumblebee resources, with possible benefits to landscape connectivity) had been accounted for, there may have been more potential to detect impacts on landscape‐scale bumblebee densities. As such, further research is required to investigate the density of solar farms needed in the landscape to make a difference to bumblebee populations at larger spatial scales.

Although the findings indicate that both solar farm management and surrounding land‐use change impact bumblebee density, they are only applicable to the legacy of existing solar farms. We show that the solar farms in operation could continue to support bumblebees in their current landscapes as they undergo land‐use changes, but the implications may differ for new solar farms deployed elsewhere. More than 90,000 ha of land across the UK may be used for solar farms by 2050 to meet Net Zero targets (based on current proportions of ground‐mounted to rooftop installations), but it is likely that the amount and location of solar farms will vary depending on policies, grid constraints and levels of future environmental sustainability (Palmer et al. 2019; Blaydes, Whyatt, et al. 2025). As such, deployment may be driven elsewhere and new solar farms might be located in different landscapes, less dominated by agriculture, which would lead to different net effects. However, strategic siting decisions for new developments could be optimised to maximise biodiversity benefits through careful placement to support landscape connectivity or to provide pollinator habitats where they are otherwise limited (Blaydes et al. 2021), and agricultural decision support tools could be used to support and streamline this decision making (Redhead et al. 2022). As the findings suggest that solar farms have a very localised impact on bumblebee densities, approaches to landscape planning should be targeted. Careful siting and management could also benefit other pollinators and other taxa such as birds (Jarčuška et al. 2024), but further research is required to better understand biodiversity responses to solar farms in both the present day and in the future.

To date, there have been no attempts to predict biodiversity responses to solar farms in the future, and few studies in other contexts focus on future land‐use effects at such high spatial resolution (Titeux et al. 2016). Studies often combine projections of future land use with species distribution models (Suzuki‐Ohno et al. 2020), which do not account for how species use habitats or the impacts of landscape connectivity. Or, studies use process‐based models focusing on individual species paired with typically simple land‐use projections with coarse landcover maps and few landcover classes (Beatty et al. 2016). Complex ecological models have been used to calculate future biodiversity consequences for offshore renewable energy developments; but, given the marine context, they have not had to account for changing land use (Warwick‐Evans et al. 2017). As such, this study may be the first to account for interactions of species with richly described futures at such a fine scale. This represents a significant development in our ability to predict future biodiversity responses to land‐use changes and habitat interventions.

Future scenarios typically focus on socioeconomics and do not always specify in detail factors that affect biodiversity, making it difficult to directly estimate biodiversity consequences. However, the methods used in this study demonstrate that it is possible to integrate biodiversity into existing future land‐use scenarios by interpreting associated narratives, downscaling future land‐use maps to account for features and microhabitats of importance to species and the application of process‐based ecological models. Models can then be used to predict biodiversity responses to future land‐use changes, with assumptions and caveats relating to the downscaling and modelling documented alongside results so that limitations and simplifications can be identified and reviewed. The approach used in this study could be easily applied to other contexts, taxa and geographic regions, providing valuable insight into how biodiversity might respond to future land‐use changes. The spatial downscaling process that enables the production of high‐resolution land‐use maps from coarser scenarios using user‐defined land‐use transitions is available as an open access ArcGIS Pro workflow and can be applied to any geographic area (Gallego et al. 2025). Coupling this downscaling approach with process‐based models, including the wider *4pop family (which simulates birds, bats, reptiles and amphibians; Gardner et al. 2024), or with similar models tailored or parameterised for the geographic region of interest, will expand understanding of the role of land‐use change on biodiversity and could support the development of effective conservation policies.

Overall, this study represents the first investigations into the roles of solar farms in future biodiversity conservation. Our results indicate that well‐managed solar farms could provide an opportunity to help protect very localized bumblebee populations against future land‐use changes occurring outside of site boundaries, for a range of levels of sustainability associated with the scenario driving land‐use change. While benefits may be limited to the local scale, this understanding helps to contextualize the role of solar farms amid future threats to pollinators and may help to ensure biodiversity is embedded in the transition to renewable energy. Solar farms should be considered as an emerging tool in conservation which could potentially deliver benefits into the future if managed appropriately.

## Author Contributions

**Hollie Blaydes:** conceptualization, data curation, formal analysis, methodology, project administration, visualization, writing – original draft, writing – review and editing. **Emma Gardner:** conceptualization, formal analysis, methodology, resources, writing – review and editing. **J. Duncan Whyatt:** conceptualization, methodology, writing – review and editing. **Simon G. Potts:** conceptualization, writing – review and editing. **Robert Dunford‐Brown:** conceptualization, writing – review and editing. **John W. Redhead:** resources, writing – review and editing. **Alona Armstrong:** conceptualization, writing – review and editing.

## Conflicts of Interest

The authors declare no conflicts of interest.

## Supporting information


**Table S1:** UK‐RCP‐SSP land use classes and their parameters, based on G2020 landcover classes.
**Table S2:** Seasonal floral cover scores (scored out of 100) with standard error for each landcover, as derived from expert opinion (Gardner et al. 2020).
**Table S3:** Floral attractiveness scores (scored out of 20) and nesting attractiveness scores (scored out of 1) with standard error for ground‐nesting bumblebees as derived from expert opinion (Gardner et al. 2020).
**Table S4:** Poll4Pop model input parameters taken from the literature showing values for bumblebees.
**Table S5:** Analysis of variance (ANOVA) and post hoc Tukey analyses results evaluating differences in foraging bumblebee density (per 100 m^2^) in 10 km landscapes surrounding solar farms managed as turf grass (*n* = 473) and meadow margins (*n* = 473) under different land use scenarios where ‘SSP1’ refers to *Sustainability*, ‘SSP2’ to *Middle of the Road* and ‘SSP5’ to *Fossil‐fuelled Development*.
**Table S6:** Analysis of variance (ANOVA) and post hoc Tukey analyses results evaluating differences in foraging bumblebee density (per 100 m^2^) in 0–500 m foraging zones surrounding solar farms managed as turf grass (*n* = 1042) and meadow margins (*n* = 1042) under different land‐use scenarios and solar farm management regimes.
**Table S7:** Analysis of variance (ANOVA) and post hoc Tukey analyses results evaluating differences in foraging bumblebee density (per 100 m^2^) inside solar farms managed as turf grass (*n* = 1042) and meadow margins (*n* = 1042) under different land‐use scenarios and solar farm management regimes. ANOVA results are displayed under the effect name.
**Figure S1:** Distributions of spatially averaged mean new bumblebee queen density (per 100 m^2^) in (a) 10 km landscapes surrounding solar farms (*n* = 473), (b) 0–500 m foraging zones surrounding solar farms (*n* = 1042) and (c) solar farms (*n* = 1042) across land‐use scenarios.
**Table S8:** Analysis of variance (ANOVA) and post hoc Tukey analyses results evaluating differences in new bumblebee queen density (per 100 m^2^) in 10 km landscapes surrounding solar farms managed as turf grass (*n* = 473) under different land‐use scenarios where ‘SSP1’ refers to *Sustainability*, ‘SSP2’ to *Middle of the Road* and ‘SSP5’ to *Fossil‐fuelled Development*. ANOVA results are displayed under the effect name.
**Table S9:** Analysis of variance (ANOVA) and post hoc Tukey analyses results evaluating differences in new bumblebee queen density (per 100 m^2^) in 0–500 m foraging zones surrounding solar farms managed as turf grass (*n* = 1042) and meadow margins (*n* = 1042) under different land‐use scenarios and solar farm management regimes.
**Table S10:** Analysis of variance (ANOVA) and post hoc Tukey analyses results evaluating differences in new bumblebee queen density (per 100 m^2^) inside solar farms managed as turf grass (*n* = 1042) and meadow margins (*n* = 1042) under different land‐use scenarios and solar farm management regimes. ANOVA results are displayed under the effect name.
**Figure S2:** Distributions of the mean (a) foraging resources present in solar farm landscapes (*n* = 473), (b) nesting resources present in solar farm landscapes (*n* = 473), (c) foraging resources present in solar farm foraging zones (*n* = 1042) and (d) nesting resources present in solar farm foraging zones (*n* = 1042) across land‐use scenarios.
**Table S11:** Analysis of variance (ANOVA) and post hoc Tukey analyses results evaluating differences in total floral resources in 10 km landscapes surrounding solar farms managed as turf grass (*n* = 473) under different land‐use scenarios where ‘SSP1’ refers to *Sustainability*, ‘SSP2’ to *Middle of the Road* and ‘SSP5’ to *Fossil‐fuelled Development*.
**Table S12:** Analysis of variance (ANOVA) and post hoc Tukey analyses results evaluating differences in total nesting resources in 10 km landscapes surrounding solar farms managed as turf grass (*n* = 473) under different land‐use scenarios where ‘SSP1’ refers to *Sustainability*, ‘SSP2’ to *Middle of the Road* and ‘SSP5’ to *Fossil‐fuelled Development*.
**Table S13:** Analysis of variance (ANOVA) and post hoc Tukey analyses results evaluating differences in total floral resources in 0–500 m foraging zones surrounding solar farms (*n* = 1042) under different land‐use scenarios where ‘SSP1’ refers to *Sustainability*, ‘SSP2’ to *Middle of the Road* and ‘SSP5’ to *Fossil‐fuelled Development*.
**Table S14:** Analysis of variance (ANOVA) and post hoc Tukey analyses results evaluating differences in total nesting resources in 0–500 m foraging zones surrounding solar farms (*n* = 1042) under different land‐use scenarios where ‘SSP1’ refers to *Sustainability*, ‘SSP2’ to *Middle of the Road* and ‘SSP5’ to *Fossil‐fuelled Development*.
**Table S15:** Generalised linear model output describing the variation in the change in foraging bumblebee density in 10 km landscapes surrounding solar farms between the present day and (A) SSP1 (*Sustainability*), (B) SSP2 (*Middle of the Road*) and (C) SSP5 (*Fossil‐fuelled Development*).
**Table S16:** Generalised linear model output describing the variation in the change in foraging bumblebee density in 0–500 m foraging zones surrounding solar farms between the present day and (A) SSP1 (*Sustainability*), (B) SSP2 (*Middle of the Road*) and (C) SSP5 (*Fossil‐fuelled Development*).
**Table S17:** Generalised linear model output describing the variation in the change in foraging bumblebee density inside solar farms between the present day and (A) SSP1 (*Sustainability*), (B) SSP2 (*Middle of the Road*) and (C) SSP5 (*Fossil‐fuelled Development*).
**Table S18:** Generalised linear model output describing the variation in the change in foraging bumblebee density inside solar farms between the present day and (A) SSP1 (*Sustainability*), (B) SSP2 (*Middle of the Road*) and (C) SSP5 (*Fossil‐fuelled Development*).
**Figure S3:** Box plots of landcover and feature area change included in generalised linear models to explain variation in foraging bumblebee density change from the present day to future scenarios where (a) and (b) show change from the present day to SSP1 (*Sustainability*), (c) and (d) to SSP2 (*Middle of the Road*) and (e) and (f) to SSP5 (*Fossil‐fuelled Development*).
**Text S1:** Overlapping landscapes.
**Text S2:** Land use transition decisions.
**Text S3:** Characterisation of arable land use types.
**Text S4:** Addition of hedgerows to landscapes.
**Text S5:** Pollinator modelling.
**Text S6:** New bumblebee queen response to land use and management scenarios.
**Text S7:** Bumblebee foraging and nesting resources across land use scenarios.

## Data Availability

The data that support the findings of this study are openly available in Dryad at https://doi.org/10.5061/dryad.8cz8w9h5g.
