# Supplementary material for: Solar Farms as Potential Future Refuges for Bumblebees
Source: Glob Chang Biol. 2025 Oct 8;31(10):e70537. doi: 10.1111/gcb.70537 (PMC12505205; doi:10.1111/gcb.70537)
Supplement: Supplementary file 1 — Table S1: UK‐RCP‐SSP land use classes and their parameters, based on G2020 landcover classes. Table S2: Seasonal floral cover scores (scored out of 100) with standard error for each landcover, as derived from expert opinion (Gardner et al. 2020). Table S3: Floral attractiveness scores (scored out of 20) and nesting attractiveness scores (scored out of 1) with standard error for ground‐nesting bumblebees as derived from expert opinion (Gardner et al. 2020). Table S4: Poll4Pop model input parameters taken from the literature showing values for bumblebees. Table S5: Analysis of variance (ANOVA) and post hoc Tukey analyses results evaluating differences in foraging bumblebee density (per 100 m2) in 10 km landscapes surrounding solar farms managed as turf grass (n = 473) and meadow margins (n = 473) under different land use scenarios where ‘SSP1’ refers to Sustainability, ‘SSP2’ to Middle of the Road and ‘SSP5’ to Fossil‐fuelled Development. Table S6: Analysis of variance (ANOVA) and post hoc Tukey analyses results evaluating differences in foraging bumblebee density (per 100 m2) in 0–500 m foraging zones surrounding solar farms managed as turf grass (n = 1042) and meadow margins (n = 1042) under different land‐use scenarios and solar farm management regimes. Table S7: Analysis of variance (ANOVA) and post hoc Tukey analyses results evaluating differences in foraging bumblebee density (per 100 m2) inside solar farms managed as turf grass (n = 1042) and meadow margins (n = 1042) under different land‐use scenarios and solar farm management regimes. ANOVA results are displayed under the effect name. Figure S1: Distributions of spatially averaged mean new bumblebee queen density (per 100 m2) in (a) 10 km landscapes surrounding solar farms (n = 473), (b) 0–500 m foraging zones surrounding solar farms (n = 1042) and (c) solar farms (n = 1042) across land‐use scenarios. Table S8: Analysis of variance (ANOVA) and post hoc Tukey analyses results evaluating differences in new bumb [file GCB-31-e70537-s001.docx]

**Supporting Information: Solar farms as potential future refuges for bumblebees**

Hollie Blaydes^1*^, Emma Gardner^2^, J Duncan Whyatt^1^, Simon G Potts^3^, Robert Dunford-Brown^2^, John W Redhead^2^ and Alona Armstrong^1,4^

^1^Lancaster Environment Centre, Library Avenue, Lancaster University, Lancaster, LA1 4YQ, UK

^2^UK Centre for Ecology and Hydrology, Wallingford, OX10 8BB, UK

3Centre for Agri-Environmental Research, School of Agriculture, Policy and Development, University of Reading, Reading, RG6 6AR, UK

^4^Energy Lancaster, Science and Technology Building, Lancaster University, Lancaster, LA1 4YF, UK

^*^corresponding author details: [h.blaydes@lancaster.ac.uk](mailto:h.blaydes@lancaster.ac.uk)

**Methods**

**Text S1:** *Overlapping landscapes*

Landscape squares with significant overlap (> 25%) were removed from the landscape sample for landscape-level analyses using a multi-step approach undertaken in R (version 2023.03.0 + 386; R Core Team, 2023). Firstly, all overlapping landscapes were identified and the percentage overlap in each case was calculated. Next, an overlapping landscape was randomly selected and if there were any landscapes that overlapped with this focal landscape by > 25%, they were removed. The maximum overlap percentage across the remaining landscape sample was then recalculated and if this was still above 25%, random landscape selection and removal of overlapping landscapes by > 25%, was repeated. This was repeated until no landscape square was overlapped by any other landscape by more than 25%.

**Text S2:** *Land use transition decisions*

The first step when downscaling UK-RCP-SSP land use maps was to identify every possible land use transition from the G2020 landcover map to the UK-RCP-SSP land use maps (24 present day landcover classes x 17 future land use classes = 408 transitions in total) and an outcome for each transition was determined. For example, if a cell in the G2020 map was classed as improved permanent grassland but in a UK-RCP-SSP map, the same cell was classed as urban, the cell in the hybrid map would transition to urban, given grassland could be developed into an urban area. In contrast, cells classed as water, beaches, moorland, salt marsh, wetland or suburban in the G2020 map did not undergo transitions, given these landcover classes were not represented in UK-RCP-SSP maps. All landcover classes in the hybrid map were represented by landcover classes from the G2020 map as these have been scored by experts in terms of their foraging and nesting value to bumblebees (Gardner et al., 2020).

In some cases, there were direct equivalent landcover classes in the G2020 and the UK-RCP-SSP maps. Where there were no direct equivalents, the G2020 landcover classes were modified to create new classes (Table S1). For example, very extensive pasture was assigned the mean scores of G2020 landcovers unimproved permanent grassland and unimproved meadow. In other cases, there were future land use classes that were relatively similar but were assigned different scores to try to reflect variation. For example, there were six woodland land use classes included in UK-RCP-SSP maps (productive native broadleaf, productive non-native broadleaf, productive conifer, productive non-native conifer, native woodland (conservation) and multifunctional mixed woodland), which varied in terms of woodland type, function and whether the woodland was made up of native or non-native species. To reflect differences in these land use classes, each were assigned different scores, where productive native broadleaf and productive non-native broadleaf were parameterised as the G2020 landcover class afforestation, productive native conifer and productive non-native conifer as coniferous woodland, native woodland (conservation) as deciduous woodland and multifunctional mixed woodland as a mean of coniferous and deciduous scores.

Most landcover class scores were consistent across scenarios, but scores for urban areas differed by scenario. In *Sustainability*, urban areas were given scores equivalent to suburban, to reflect future “green cities” described in associated storylines. In *Middle of the Road*, urban areas were given a score that was the mean of urban and suburban, to reflect an intermediate future and in *Fossil-fuelled Development*, urban areas were given the score for urban, representing areas of low floral cover and attractiveness for bumblebees.

**Table S1.** UK-RCP-SSP land use classes and their parameters, based on G2020 landcover classes. “SSP1” refers to *Sustainability*, “SSP2” to *Middle of the Road* and “SSP5” to *Fossil-fuelled Development*.

| **Land use class** | **Parameters** |
| --- | --- |
| Unmanaged | Equal to present day landcover “Scrub”. |
| Bioenergy | Equal to present day landcover “Cereal”. |
| Extensive pasture | Equal to present day landcover “Unimproved permanent grassland”. |
| Intensive pasture | Equal to present day landcover “Improved permanent grassland”. |
| Multifunctional mixed woodland | Mean of the parameters of present day landcovers “Coniferous woodland” and “Deciduous woodland”. |
| Native woodland (conservation) | Equal to present day landcover “Deciduous woodland”. |
| Productive native broadleaf | Equal to present day landcover “Afforestation”. |
| Productive native conifer | Equal to present day landcover “Coniferous woodland”. |
| Productive non-native broadleaf | Equal to present day landcover “Afforestation”. |
| Productive non-native conifer | Equal to present day landcover “Coniferous woodland”. |
| Very extensive pasture | Mean of the parameters of present day landcovers “Unimproved permanent grassland” and “Unimproved meadow”. |
| Future urban | In SSP1, equal to the present day landcover “Suburban”. In SSP2, mean of the parameters of present day landcovers “Suburban” and “Urban”. In SSP5, equal to the present day landcover “Urban”. |
| Agroforestry-fallow | The weighted mean of the parameters of the present day landcover (“Fallow”; 80%) and “Orchards” (20%). |
| Agroforestry-grass ley | The weighted mean of the parameters of the present day landcover (“Grass ley”; 80%) and “Orchards” (20%). |
| Agroforestry-improved meadow | The weighted mean of the parameters of the present day landcover (“Improved meadow”; 80%) and “Orchards” (20%). |
| Agroforestry-improved permanent grassland | The weighted mean of the parameters of the present day landcover (“Improved permanent grassland”; 80%) and “Orchards” (20%). |
| Agroforestry-field beans | The weighted mean of the parameters of the present day landcover (“Field beans”; 80%) and “Orchards” (20%). |
| Agroforestry-cereal | The weighted mean of the parameters of the present day landcover (“Cereal”; 80%) and “Orchards” (20%). |
| Agroforestry-oilseed rape | The weighted mean of the parameters of the present day landcover (“Oilseed rape”; 80%) and “Orchards” (20%). |
| Agroforestry-strawberry | The weighted mean of the parameters of the present day landcover (“Strawberry/raspberry (open)”; 80%) and “Orchards” (20%). |
| Agroforestry-vegetables | The weighted mean of the parameters of the present day landcover (“Vegetables”; 80%) and “Orchards” (20%). |

**Text S3:** *Characterisation of arable land use types*

*Crop composition*

Specific crops were assigned to “intensive”, “extensive” and “sustainable” arable land parcels from the crop types included in the G2020 map, based on arable type descriptions (Redhead et al., 2020) and common crop rotations identified across the UK (Upcott et al., 2023). Temporal crop rotations were represented spatially by assigning certain proportions of arable field parcels to individual crop types within the 10 km landscapes centred on solar farms. For instance, we assume that a rotation that is 75% cereal and 25% oilseed rape means that, in a given year, 75% of the fields in the landscape would be assigned to cereal and 25% to oilseed rape. Crop landcover classes attributed to the arable land use types retained the floral and nesting scores assigned by pollinator experts.

*Depressing crop floral cover scores*

Floral cover scores for intensive crops in *Fossil-fuelled Development* were depressed to reflect intensive management practices, such as the spraying of agrochemicals, that would remove flowering non-crop species and reduce foraging resources for bumblebees. Specifically, floral cover scores for mass-flowering crops were given a score of zero outside of late spring (when the crop itself is flowering) and non-flowering crops were assigned a score of zero floral cover in all seasons.

*Setting arable field margin widths*

Field margins were added to arable fields, represented by the “grassy field margins” landcover class in G2020, whereby widths differed depending on the arable intensity and the land use scenario. Field margins in intensive arable fields were generally set to 1 m wide to represent attempts to farm to the very edge of fields (Gardner et al., 2021). However, in *Sustainability*, intensive arable fields were given 4 m margins as intensive in this scenario was assumed to mean “ecologically intensive” i.e. wide margins and other nature-based approaches are used to intensify agriculture. Margins in sustainable arable fields were set to 4 m wide across all scenarios, based on UK government guidance for Countryside Stewardship grants (UK Government, 2023a). In extensive arable fields, margins were set to 2 m, representing an intermediate between intensive and sustainable.

Arable field margins were generated in R (R Core Team, 2023) using the packages “exactextractr” (Baston, 2023), “raster” (Hijmans, 2023a) and “sf” (Pebesma, 2023a). The output was a 0/1 raster based on each of the 10 x 10 km landscapes (*n* = 1,042, for each scenario) where field margins have a value of one and the rest of the square a value of zero.

*Assigning amounts of floral rich habitat*

Flower patches were added to eligible arable land parcels managed as “sustainable arable” in *Sustainability*, *Middle of the Road* and *Fossil-fuelled Development* and to “ecologically intensive arable” in *Sustainability*, since these are a common feature of sustainably managed farmland and ecological intensification approaches (Ouvrard et al., 2018). Flower patches represent areas within farmland managed specifically to provide resources for pollinators (and other wildlife) and were represented by the “unimproved meadow” landcover class due to high floral cover and attractiveness scores (Tables S13 and S14). Five patches of 0.375 ha were generated per 100 ha inside eligible parcels based on pollinator habitat creation guidance (Nowakowski and Pywell, 2016).

Flower patches were generated in R (R Core Team, 2023) using the packages “exactextractr” (Baston, 2023), ‘lwgeom’ (Pebesma, 2023b), “raster” (Hijmans, 2023a), “sf” (Pebesma, 2023a) and ‘terra’ (Hijmans, 2023b).

**Text S4:** *Addition of hedgerows to landscapes*

Hedgerows were added to all solar farm landscapes and in the present day were represented by the UKCEH woody linear feature framework (Schofield et al., 2016). In future landscapes, total hedgerow length was based on the present day but varied across scenarios to align with associated narratives. Specifically, a 40% increase in total hedgerow length represented the meeting of ambitious targets set by the Climate Change Committee in *Sustainability* (CCC, 2018). In *Middle of the Road*, less ambitious government targets are met and there is a 16.9% increase in total hedgerow length from the present day (UK Government, 2023b). In *Fossil-fuelled Development*, hedgerow total length is equal to the present day. Individual hedgerow length was randomly chosen between 20 – 1,074 m in length, based on minimum hedgerow length definitions from the UK Government (UK Government, 2007) and the maximum length of a hedgerow in the UKCEH woody linear feature framework (Schofield et al., 2016).

Hedgerow width also differed across scenarios to reflect expected use of agri-environment scheme management options, where hedgerow width was set to 5 m in *Sustainability* and to 2.5 m in the present day, *Middle of the Road* and *Fossil-fuelled Development* scenarios (Image et al., 2022).

Hedgerows were generated in R (R Core Team, 2023) using packages “exactextractr” (Baston, 2023), “lwgeom” (Pebesma, 2023b), “raster” (Hijmans, 2023a), “sf” (Pebesma, 2023a) and “terra” (Hijmans, 2023b). Any land parcel classed as arable, grassland or agroforestry was eligible for hedgerow creation at the edges and the output was a 0/1 raster based on each of the 10 x 10 km landscapes (*n* = 1,042, for each scenario) where hedgerows have a value of one and the rest of the square a value of zero.

**Text S5:** *Pollinator modelling*

Floral cover scores, floral attractiveness and nesting attractiveness scores used with landcovers included in the G2020 map are presented in Tables S2 and S3, respectively. For landcovers present in UK-RCP-SSP land use maps, existing expert scores for G2020 landcovers were assigned (Table S1). In some cases, scores were directly equal to an existing present day landcover but in other cases, mean values were calculated across existing landcover scores (Table S1). Weighted mean proportions for agroforestry classes were derived from the literature (Image et al., 2023). The present day landcover “orchards” was used to represent fruit trees within agroforestry classes, given fruit trees were the most common group of trees used in agroforestry according to an unpublished dataset (personal communication with M.Image and T.Staton). Parameters for landcover classes generally remained the same across all scenarios (present day, *Sustainability*, *Middle of the Road* and *Fossil-fuelled Development*), apart from new “urban” areas (referred to as “future urban”), where scores changed depending on the scenario based on descriptions presented in associated narratives (Brown et al., 2022). For more detail about the expert-derived scores, see Gardner et al. (2020).

**Table S2.** Seasonal floral cover scores (scored out of 100) with standard error for each landcover, as derived from expert opinion (Gardner et al., 2020). “Future urban” parameters changed depending on the future scenario, but here *Sustainability* parameters are presented. Adapted from Blaydes et al., (2022).

|  | **Early spring** | **Late spring** | **Summer** |
| --- | --- | --- | --- |
| **Basic landcover** |  |  |  |
| Water | 0.0 ± 0.0 | 0.0 ± 0.0 | 0.0 ± 0.0 |
| Afforestation | 9.8 ± 2.7 | 9.8 ± 2.7 | 33.8 ± 9.0 |
| Beaches, sand dune or plane | 7.9 ± 1.9 | 7.9 ± 1.9 | 41.7 ± 8.0 |
| Coniferous woodland | 3.3 ± 1.4 | 3.3 ± 1.4 | 13.5 ± 4.8 |
| Deciduous woodland | 16.4 ± 2.4 | 16.4 ± 2.4 | 44.7 ± 6.6 |
| Fallow | 13.7 ± 2.8 | 13.7 ± 2.8 | 47.5 ± 7.4 |
| Suburban | 24.9 ± 3.4 | 24.9 ± 3.4 | 75.9 ± 4.7 |
| Grass ley | 6.3 ± 2.3 | 6.3 ± 2.3 | 21.9 ± 6.9 |
| Improved meadow | 10.4 ± 2.1 | 10.4 ± 2.1 | 47.2 ± 4.7 |
| Improved permanent grassland | 8.7 ± 2.4 | 8.7 ± 2.4 | 28.1 ± 4.4 |
| Moorland | 8.2 ± 1.7 | 8.2 ± 1.7 | 48.9 ± 7.7 |
| Salt marsh | 5.5 ± 2.7 | 5.5 ± 2.7 | 33.1 ± 14.1 |
| Scrub | 13.5 ± 2.3 | 13.5 ± 2.3 | 45.9 ± 5.4 |
| Unimproved meadow | 12.8 ± 2.4 | 12.8 ± 2.4 | 72.1 ± 7.0 |
| Unimproved permanent grassland | 11.3 ± 2.0 | 11.3 ± 2.0 | 64.2 ± 10.1 |
| Wetland | 5.3 ± 1.1 | 5.3 ± 1.1 | 44.4 ± 9.0 |
| Berries | 0.0 ± 0.0 | 27.2 ± 6.9 | 47.0 ± 9.1 |
| Field beans | 0.0 ± 0.0 | 21.5 ± 7.1 | 27.0 ± 7.0 |
| Cereal | 3.1 ± 1.3 | 3.1 ± 1.3 | 10.5 ± 3.0 |
| Oilseed rape | 0.0 ± 0.0 | 29.5 ± 2.6 | 19.8 ± 6.4 |
| Orchards | 31.8 ± 4.9 | 0.0 ± 0.0 | 32.6 ± 10.3 |
| Strawberry/raspberry (polytunnel) | 0.0 ± 0.0 | 48.4 ± 11.7 | 61.6 ± 11.8 |
| Strawberry/raspberry (open) | 0.0 ± 0.0 | 10.0 ± 3.7 | 61.4 ± 11.4 |
| Vegetables | 2.5 ± 2.4 | 2.5 ± 2.4 | 37.1 ± 15.1 |
| Urban | 0.0 ± 0.0 | 0.0 ± 0.0 | 0.0 ± 0.0 |
| Unmanaged | 13.5 ± 2.3 | 13.5 ± 2.3 | 45.9 ± 5.4 |
| Bioenergy | 3.1 ± 1.3 | 3.1 ± 1.3 | 10.5 ± 3.0 |
| Extensive pasture | 11.3 ± 2.0 | 11.3 ± 2.0 | 64.2 ± 10.1 |
| Intensive pasture | 8.7 ± 2.4 | 8.7 ± 2.4 | 28.1 ± 4.4 |
| Multifunctional mixed woodland | 9.9 ± 1.4 | 9.9 ± 1.4 | 29.1 ± 4.1 |
| Native woodland (conservation) | 16.4 ± 2.4 | 16.4 ± 2.4 | 44.7 ± 6.6 |
| Productive native broadleaf | 9.8 ± 2.7 | 9.8 ± 2.7 | 33.8 ± 9.0 |
| Productive native conifer | 3.3 ± 1.4 | 3.3 ± 1.4 | 13.5 ± 4.8 |
| Productive non-native broadleaf | 9.8 ± 2.7 | 9.8 ± 2.7 | 33.8 ± 9.0 |
| Productive non-native conifer | 3.3 ± 1.4 | 3.3 ± 1.4 | 13.5 ± 4.8 |
| Very extensive pasture | 12 ± 1.6 | 12 ± 1.6 | 68.1 ± 6.1 |
| Future urban | 24.9 ± 3.4 | 24.9 ± 3.4 | 75.9 ± 4.7 |
| Agroforestry-fallow | 17.3 ± 4.5 | 10.9 ± 2.3 | 44.5 ± 10.1 |
| Agroforestry-grass ley | 11.4 ± 4.3 | 5.0 ± 1.8 | 24.1 ± 9.9 |
| Agroforestry-improved meadow | 14.7 ± 4.2 | 8.4 ± 1.7 | 44.3 ± 9.1 |
| Agroforestry-improved permanent grassland | 13.3 ± 4.3 | 7 ± 1.9 | 29.0 ± 9.0 |
| Agroforestry-field beans | 6.4 ± 3.9 | 17.2 ± 5.7 | 28.1 ± 10.0 |
| Agroforestry-cereal | 8.9 ± 4 | 2.5 ± 1.1 | 15.0 ± 8.6 |
| Agroforestry-oilseed rape | 6.4 ± 3.9 | 23.6 ± 2.1 | 22.3 ± 9.7 |
| Agroforestry-strawberry | 6.4 ± 3.9 | 8.0 ± 3.0 | 55.7 ± 12.3 |
| Agroforestry-vegetables | 8.4 ± 4.4 | 2.0 ± 2.0 | 36.2 ± 14.6 |
| **Solar farm landcover** |  |  |  |
| Baseline | 0.0 ± 0.0 | 0.0 ± 0.0 | 0.0 ± 0.0 |
| Turf grass | 8.7 ± 2.4 | 8.7 ± 2.4 | 28.1 ± 4.4 |
| Meadow margins | 12.8 ± 2.4 | 12.8 ± 2.4 | 72.1 ± 7.0 |

**Table S3.** Floral attractiveness scores (scored out of 20) and nesting attractiveness scores (scored out of 1) with standard error for ground-nesting bumblebees as derived from expert opinion (Gardner et al., 2020). Adapted from Blaydes et al. (2022).

|  | **Floral attractiveness** | **Nesting attractiveness** |
| --- | --- | --- |
| **Basic landcover** |  |  |
| Water | 0.0 ± 0.0 | 0.0 ± 0.0 |
| Afforestation | 7.9 ± 1.8 | 0.5 ± 0.0 |
| Beaches, sand dune or plane | 11.1 ± 2.1 | 0.3 ± 0.1 |
| Coniferous woodland | 3.9 ± 1.1 | 0.4 ± 0.1 |
| Deciduous woodland | 12.0 ± 1.1 | 0.6 ± 0.1 |
| Fallow | 12.1 ± 1.4 | 0.6 ± 0.1 |
| Suburban | 17.6 ± 0.9 | 0.8 ± 0.0 |
| Grass ley | 5.0 ± 1.2 | 0.3 ± 0.1 |
| Improved meadow | 7.6 ± 1.8 | 0.4 ± 0.1 |
| Improved permanent grassland | 4.6 ± 1.1 | 0.2 ± 0.1 |
| Moorland | 14.8 ± 1.6 | 0.5 ± 0.0 |
| Salt marsh | 9.5 ± 1.7 | 0.1 ± 0.1 |
| Scrub | 15.3 ± 1.6 | 0.7 ± 0.1 |
| Unimproved meadow | 15.9 ± 1.6 | 0.7 ± 0.1 |
| Unimproved permanent grassland | 14.1 ± 1.9 | 0.5 ± 0.1 |
| Wetland | 10.5 ± 1.0 | 0.2 ± 0.1 |
| Berries | 15.5 ± 1.0 | 0.3 ± 0.1 |
| Field beans | 16.8 ± 0.9 | 0.3 ± 0.1 |
| Cereal | 1.0 ± 0.6 | 0.1 ± 0.1 |
| Oilseed rape | 17.3 ± 0.6 | 0.2 ± 0.1 |
| Orchards | 16.7 ± 0.6 | 0.6 ± 0.1 |
| Strawberry/raspberry (polytunnel) | 11.8 ± 2.5 | 0.1 ± 0.1 |
| Strawberry/raspberry (open) | 16.3 ± 0.9 | 0.4 ± 0.1 |
| Vegetables | 6.3 ± 2.5 | 0.2 ± 0.1 |
| Urban | 0.0 ± 0.0 | 0.0 ± 0.0 |
| Unmanaged | 15.3 ± 1.6 | 0.7 ± 0.1 |
| Bioenergy | 1.0 ± 0.6 | 0.1 ± 0.1 |
| Extensive pasture | 14.1 ± 1.9 | 0.5 ± 0.1 |
| Intensive pasture | 4.6 ± 1.1 | 0.2 ± 0.1 |
| Multifunctional mixed woodland | 7.9 ± 0.8 | 0.5 ± 0.0 |
| Native woodland (conservation) | 12 ± 1.1 | 0.6 ± 0.1 |
| Productive native broadleaf | 7.9 ± 1.8 | 0.5 ± 0.0 |
| Productive native conifer | 3.9 ± 1.1 | 0.4 ± 0.1 |
| Productive non-native broadleaf | 7.9 ± 1.8 | 0.5 ± 0.0 |
| Productive non-native conifer | 3.9 ± 1.1 | 0.4 ± 0.1 |
| Very extensive pasture | 15.0 ± 1.2 | 0.6 ± 0.1 |
| Future urban | 17.6 ± 0.9 | 0.8 ± 0.0 |
| Agroforestry-fallow | 13.0 ± 1.2 | 0.6 ± 0.1 |
| Agroforestry-grass ley | 7.4 ± 1.1 | 0.4 ± 0.1 |
| Agroforestry-improved meadow | 9.4 ± 1.5 | 0.4 ± 0.1 |
| Agroforestry-improved permanent grassland | 7.0 ± 1.0 | 0.3 ± 0.1 |
| Agroforestry-field beans | 16.8 ± 0.8 | 0.3 ± 0.1 |
| Agroforestry-cereal | 4.2 ± 0.7 | 0.2 ± 0.1 |
| Agroforestry-oilseed rape | 17.2 ± 0.7 | 0.3 ± 0.1 |
| Agroforestry-strawberry | 16.3 ± 0.8 | 0.4 ± 0.1 |
| Agroforestry-vegetables | 8.3 ± 2.1 | 0.2 ± 0.1 |
| **Solar farm landcover** |  |  |
| Baseline | 0.0 ± 0.0 | 0.0 ± 0.0 |
| Turf grass | 4.6 ± 1.1 | 0.2 ± 0.1 |
| Meadow margins | 15.9 ± 1.6 | 0.7 ± 0.1 |

**Table S4.** Poll4Pop model input parameters taken from the literature showing values for bumblebees. Reproduced from Gardner et al. (2020) and Blaydes et al., (2022).

| **Parameter** | **Description** | **Unit** | **Value** |
| --- | --- | --- | --- |
| *n* _max_ | Number of nests in a cell of maximum nesting quality | nests/ha | 19 |
| *β* | Mean dispersal distance for foraging | m | 530 |
| 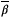 | Mean dispersal distance to new nesting sites | m | 1000 |
| *a_w_* | Median of the growth rate for workers | - | 100 |
| *b_w_* | Steepness of the growth rate for workers | - | 200 |
| *a_q_* | Median of the growth rate for reproductive females | - | 15,000 |
| *b_q_* | Steepness of the growth rate for reproductive females | - | 30,000 |
| *w* _max_ | Maximum number of workers produced by a reproductive female | - | 600 |
| *q* _max_ | Maximum number of new reproductive females produced | - | 160 |
| *p_w_* | Fraction of foraging workers | - | 0.5 |

**Results**

*Foraging bumblebee response to land use and management scenarios*

**Table S5.** Analysis of variance (ANOVA) and post-hoc Tukey analyses results evaluating differences in foraging bumblebee density (per 100 m^2^) in 10 km landscapes surrounding solar farms managed as turf grass (*n* = 473) and meadow margins (*n* = 473) under different land use scenarios where “SSP1” refers to *Sustainability*, “SSP2” to *Middle of the Road* and “SSP5” to *Fossil-fuelled Development*. ANOVA results are displayed under the effect name. “Group 1” and “Group 2” refer to the treatment groups being compared in Tukey analyses and “CI” refers to “confidence intervals”. Bold values indicate a significant difference at the P < 0.05 level between groups. Foraging bumblebee density values were transformed using Box-Cox methods, where λ = 0.34, to meet test assumptions of normality.

| **Effect** | **Group 1** | **Group 2** | **Estimate** | **CI** | **P** |
| --- | --- | --- | --- | --- | --- |
| **Scenario** (when solar farms are managed as **turf grass**)  F(3, 1888) = 35.0, P < 0.001 | Present | SSP1 | 0.14 | 0.10, 0.18 | **< 0.001** |
|  | Present | SSP2 | 0.10 | 0.06, 0.14 | **< 0.001** |
|  | Present | SSP5 | 0.04 | -0.001, 0.08 | 0.06 |
|  | SSP1 | SSP2 | -0.04 | -0.08, 0.0005 | 0.05 |
|  | SSP1 | SSP5 | -0.10 | -0.14, -0.06 | **< 0.001** |
|  | SSP2 | SSP5 | -0.07 | -0.11, -0.03 | **< 0.001** |
| **Scenario** (when solar farms are managed with **meadow margins**)  F(3, 1888) = 35.1, P < 0.001 | Present | SSP1 | 0.14 | 0.10, 0.18 | **< 0.001** |
|  | Present | SSP2 | 0.10 | 0.06, 0.14 | **< 0.001** |
|  | Present | SSP5 | 0.04 | -0.001, 0.08 | 0.06 |
|  | SSP1 | SSP2 | -0.04 | -0.08, 0.0005 | 0.05 |
|  | SSP1 | SSP5 | -0.10 | -0.14, -0.06 | **< 0.001** |
|  | SSP2 | SSP5 | -0.07 | -0.11, -0.03 | **< 0.001** |
| **Management**  F(1, 3782) = 0.004, P < 0.95 | Turf grass | Meadow margins | 0.0005 | 0.00, 1.00 | 0.95 |

**Table S6.** Analysis of variance (ANOVA) and post-hoc Tukey analyses results evaluating differences in foraging bumblebee density (per 100 m^2^) in 0 – 500 m foraging zones surrounding solar farms managed as turf grass (*n* = 1,042) and meadow margins (*n* = 1,042) under different land use scenarios and solar farm management regimes. ANOVA results are displayed under the effect name. “Group 1” and “Group 2” refer to the treatment groups being compared in Tukey analyses and “CI” refers to “confidence intervals”. “SSP1” refers to *Sustainability*, “SSP2” to *Middle of the Road* and “SSP5” to *Fossil-fuelled Development.* Bold values indicate a significant difference at the P < 0.05 level between groups. Foraging bumblebee density values were transformed using Box-Cox methods, where λ = 0.30, to meet test assumptions of normality.

| **Effect** | **Group 1** | **Group 2** | **Estimate** | **CI** | **P** |
| --- | --- | --- | --- | --- | --- |
| **Scenario**  (when solar farms are managed as **turf grass**)  F(3, 4164) = 51.1, P < 0.001 | Present | SSP1 | 0.66 | 0.51, 0.81 | **< 0.001** |
|  | Present | SSP2 | 0.48 | 0.33, 0.62 | **< 0.001** |
|  | Present | SSP5 | 0.25 | 0.11, 0.40 | **< 0.001** |
|  | SSP1 | SSP2 | -0.18 | -0.33, -0.04 | **0.006** |
|  | SSP1 | SSP5 | -0.41 | -0.56, -0.27 | **< 0.001** |
|  | SSP2 | SSP5 | -0.23 | -0.37, -0.08 | **< 0.001** |
| **Scenario** (when solar farms are managed with **meadow margins**)  F(3, 4164) = 51.2, P < 0.001 | Present | SSP1 | 0.65 | 0.51, 0.80 | **< 0.001** |
|  | Present | SSP2 | 0.47 | 0.33, 0.62 | **< 0.001** |
|  | Present | SSP5 | 0.25 | 0.10, 0.39 | **< 0.001** |
|  | SSP1 | SSP2 | -0.18 | -0.33, -0.04 | **0.007** |
|  | SSP1 | SSP5 | -0.41 | -0.56, -0.27 | **< 0.001** |
|  | SSP2 | SSP5 | -0.23 | -0.37, -0.08 | **< 0.001** |
| **Management**  F(1, 8334) = 816, P < 0.001 | Turf grass | Meadow margins | 0.03 | -0.03, 0.08 | 0.33 |

**Table S7.** Analysis of variance (ANOVA) and post-hoc Tukey analyses results evaluating differences in foraging bumblebee density (per 100 m^2^) inside solar farms managed as turf grass (*n* = 1,042) and meadow margins (*n* = 1,042) under different land use scenarios and solar farm management regimes. ANOVA results are displayed under the effect name. “Group 1” and “Group 2” refer to the treatment groups being compared in Tukey analyses and “CI” refers to “confidence intervals”. “SSP1” refers to *Sustainability*, “SSP2” to *Middle of the Road* and “SSP5” to *Fossil-fuelled Development.* Bold values indicate a significant difference at the P < 0.05 level between groups. Foraging bumblebee density values were transformed using Box-Cox methods, where λ = 0.22, to meet test assumptions of normality.

| **Effect** | **Group 1** | **Group 2** | **Estimate** | **CI** | **P** |
| --- | --- | --- | --- | --- | --- |
| **Scenario**  (when solar farms are managed as **turf grass**)  F(3, 4164) = 23.3, P < 0.001 | Present | SSP1 | 0.30 | 0.20, 0.39 | **< 0.001** |
|  | Present | SSP2 | 0.21 | 0.11, 0.30 | **< 0.001** |
|  | Present | SSP5 | 0.22 | 0.12, 0.31 | **< 0.001** |
|  | SSP1 | SSP2 | -0.09 | -0.19, 0.006 | 0.08 |
|  | SSP1 | SSP5 | -0.08 | -0.17, 0.02 | 0.15 |
|  | SSP2 | SSP5 | 0.01 | -0.09, 0.11 | 0.99 |
| **Scenario** (when solar farms are managed with **meadow margins**)  F(3, 4164) = 17.9, P < 0.001 | Present | SSP1 | 0.36 | 0.23, 0.49 | **< 0.001** |
|  | Present | SSP2 | 0.26 | 0.13, 0.39 | **< 0.001** |
|  | Present | SSP5 | 0.28 | 0.14, 0.41 | **< 0.001** |
|  | SSP1 | SSP2 | -0.10 | -0.23, 0.03 | 0.21 |
|  | SSP1 | SSP5 | -0.08 | -0.22, 0.05 | 0.37 |
|  | SSP2 | SSP5 | 0.02 | -0.12, 0.15 | 0.98 |
| **Management**  F(1, 8334) = 2709, P < 0.001 | Turf grass | Meadow margins | 1.18 | 1.14, 1.23 | **< 0.001** |

*New bumblebee queen response to land use and management scenarios*

**Text S6:** *New bumblebee queen response to land use and management scenarios*

At the landscape and foraging zone scale, new bumblebee queen density was greater under *Sustainability* and *Middle of the Road* scenarios, compared to the present day and *Fossil-fuelled Development,* but solar farm management had no effect (Figure S1, Tables S8 and S9). In contrast, management was the strongest driver in new bumblebee queen densities at the solar farm scale, where the density of new queens was always higher in solar farms with meadow margins compared to solar farms managed as turf grass (Figure S1, Table S10). Land use scenario also had an impact at this scale, but the effect depended on solar farm management. When solar farms were managed as turf grass, the density of new bumblebee queens was greatest under *Sustainability*, followed by *Middle of the Road*, the present day and *Fossil-fuelled Development* (Figure S1, Table S10). However, in solar farms with meadow margins, new bumblebee queen density was greatest under *Sustainability*, with no significant difference between the present day and *Middle of the Road* and *Fossil-fuelled Development* (Figure S1, Table S10).

**
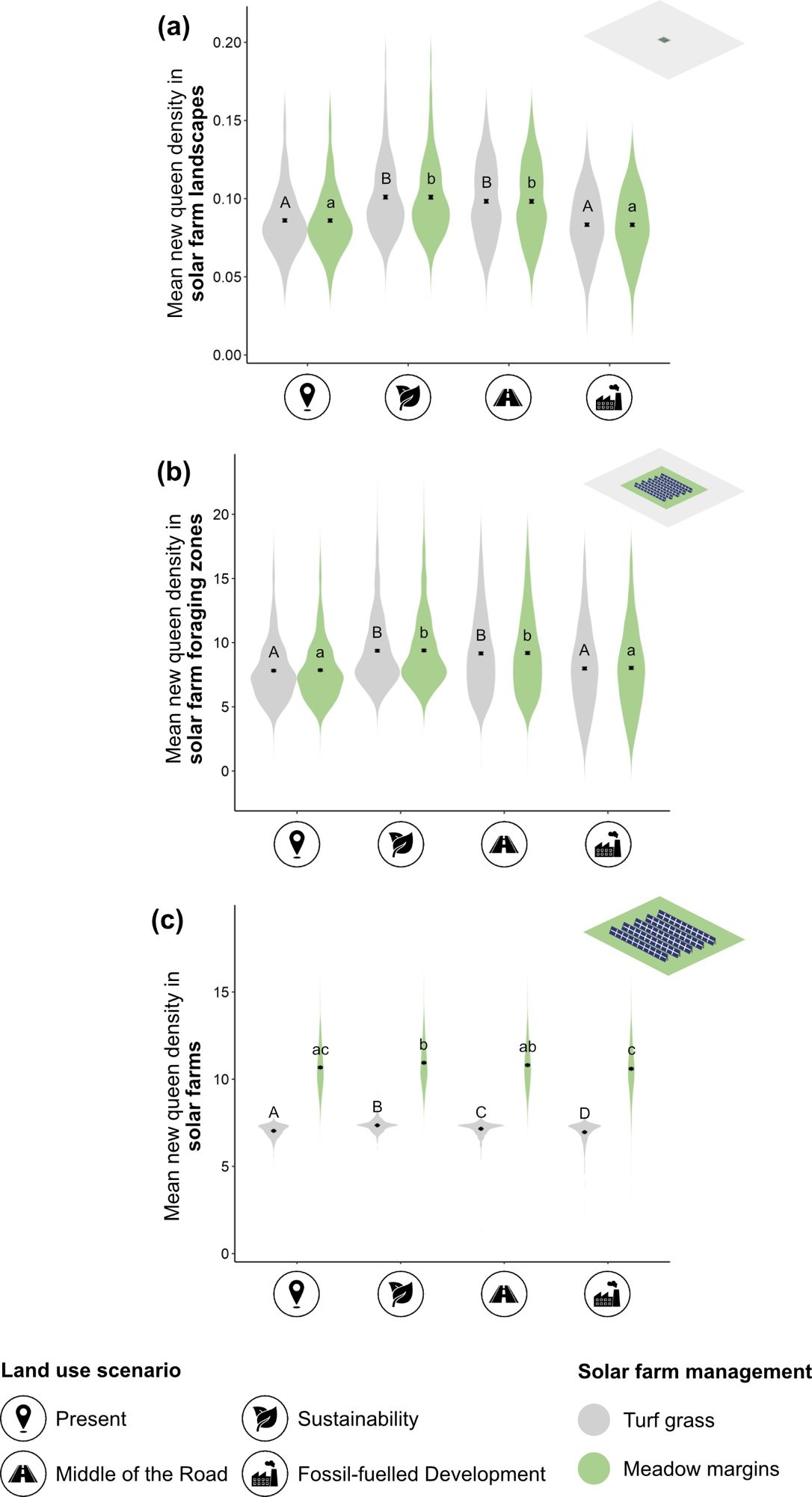
**

**Figure S1.** Distributions of spatially-averaged mean new bumblebee queen density (per 100 m^2^) in (a) 10 km landscapes surrounding solar farms (*n* = 473), (b) 0 – 500 m foraging zones surrounding solar farms (*n* = 1,042) and (c) solar farms (*n* = 1,042) across land use scenarios. Black points show the sample-level mean and error bars represent the standard error on this sample-level mean. Within each plot, points that share letters are not significantly different at the P < 0.05 level according to ANOVA and Tukey post-hoc analyses. Upper-case letters present the results of ANOVA and Tukey analyses relating to solar farms managed as turf grass (grey) and lower-case letters present results relating to solar farms managed with meadow margins (green). Land use scenario icons were reproduced from the Noun Project (<https://thenounproject.com>).

**Table S8.** Analysis of variance (ANOVA) and post-hoc Tukey analyses results evaluating differences in new bumblebee queen density (per 100 m^2^) in 10 km landscapes surrounding solar farms managed as turf grass (*n* = 473) under different land use scenarios where “SSP1” refers to *Sustainability*, “SSP2” to *Middle of the Road* and “SSP5” to *Fossil-fuelled Development*. ANOVA results are displayed under the effect name. “Group 1” and “Group 2” refer to the treatment groups being compared in Tukey analyses and “CI” refers to “confidence intervals”. Bold values indicate a significant difference at the P < 0.05 level between groups. New bumblebee queen density values had a normal distribution and therefore did not require transformation to meet test assumptions.

| **Effect** | **Group 1** | **Group 2** | **Estimate** | **CI** | **P** |
| --- | --- | --- | --- | --- | --- |
| **Scenario** (when solar farms are managed as **turf grass**)  F(3, 1888) = 70.2, P < 0.001 | Present | SSP1 | 0.01 | 0.01, 0.02 | **< 0.001** |
|  | Present | SSP2 | 0.01 | 0.008, 0.016 | **< 0.001** |
|  | Present | SSP5 | -0.003 | -0.007, -0.001 | 0.26 |
|  | SSP1 | SSP2 | -0.003 | -0.006, 0.001 | 0.26 |
|  | SSP1 | SSP5 | -0.02 | -0.02, -0.01 | **< 0.001** |
|  | SSP2 | SSP5 | -0.01 | -0.02, -0.01 | **< 0.001** |
| **Scenario** (when solar farms are managed with **meadow margins**)  F(3, 1888) = 70.2, P < 0.001 | Present | SSP1 | 0.01 | 0.01, 0.02 | **< 0.001** |
|  | Present | SSP2 | 0.01 | 0.008, 0.016 | **< 0.001** |
|  | Present | SSP5 | -0.003 | -0.007, -0.001 | 0.26 |
|  | SSP1 | SSP2 | -0.003 | -0.006, 0.001 | 0.27 |
|  | SSP1 | SSP5 | -0.02 | -0.02, -0.01 | **< 0.001** |
|  | SSP2 | SSP5 | -0.01 | -0.02, -0.01 | **< 0.001** |
| **Management**  F(1, 3782) = 0.007, P = 0.94 | Turf grass | Meadow margins | 0.0006 | 0.00, 1.00 | 0.94 |

**Table S9.** Analysis of variance (ANOVA) and post-hoc Tukey analyses results evaluating differences in new bumblebee queen density (per 100 m^2^) in 0 – 500 m foraging zones surrounding solar farms managed as turf grass (*n* = 1,042) and meadow margins (*n* = 1,042) under different land use scenarios and solar farm management regimes. ANOVA results are displayed under the effect name. “Group 1” and “Group 2” refer to the treatment groups being compared in Tukey analyses and “CI” refers to “confidence intervals”. “SSP1” refers to *Sustainability*, “SSP2” to *Middle of the Road* and “SSP5” to *Fossil-fuelled Development.* Bold values indicate a significant difference at the P < 0.05 level between groups. New bumblebee queen density values had a normal distribution and therefore did not require transformation to meet test assumptions.

| **Effect** | **Group 1** | **Group 2** | **Estimate** | **CI** | **P** |
| --- | --- | --- | --- | --- | --- |
| **Scenario**  (when solar farms are managed as **turf grass**)  F(3, 4164) = 76.4, P < 0.001 | Present | SSP1 | 1.6 | 1.2, 1.9 | **< 0.001** |
|  | Present | SSP2 | 1.3 | 1.0, 1.7 | **< 0.001** |
|  | Present | SSP5 | 0.2 | -0.2, 0.5 | 0.56 |
|  | SSP1 | SSP2 | -0.2 | -0.5, 0.1 | 0.36 |
|  | SSP1 | SSP5 | -1.4 | -1.7, -1.1 | **< 0.001** |
|  | SSP2 | SSP5 | -1.2 | -1.5, -0.8 | **< 0.001** |
| **Scenario** (when solar farms are managed with **meadow margins**)  F(3, 4164) = 75.9, P < 0.001 | Present | SSP1 | 1.5 | 1.2, 1.9 | **< 0.001** |
|  | Present | SSP2 | 1.3 | 1.0, 1.7 | **< 0.001** |
|  | Present | SSP5 | 0.2 | -0.2, 0.5 | 0.54 |
|  | SSP1 | SSP2 | -0.2 | -0.5, 0.1 | 0.39 |
|  | SSP1 | SSP5 | -1.4 | -1.7, -1.0 | **< 0.001** |
|  | SSP2 | SSP5 | -1.2 | -1.5, -0.8 | **< 0.001** |
| **Management**  F(1, 8334) = 0.26, P = 0.61 | Turf grass | Meadow margins | 0.03 | -0.09, 0.16 | 0.61 |

**Table S10.** Analysis of variance (ANOVA) and post-hoc Tukey analyses results evaluating differences in new bumblebee queen density (per 100 m^2^) inside solar farms managed as turf grass (*n* = 1,042) and meadow margins (*n* = 1,042) under different land use scenarios and solar farm management regimes. ANOVA results are displayed under the effect name. “Group 1” and “Group 2” refer to the treatment groups being compared in Tukey analyses and “CI” refers to “confidence intervals”. “SSP1” refers to *Sustainability*, “SSP2” to *Middle of the Road* and “SSP5” to *Fossil-fuelled Development.* Bold values indicate a significant difference at the P < 0.05 level between groups. New bumblebee queen density values had a normal distribution and therefore did not require transformation to meet test assumptions.

| **Effect** | **Group 1** | **Group 2** | **Estimate** | **CI** | **P** |
| --- | --- | --- | --- | --- | --- |
| **Scenario**  (when solar farms are managed as **turf grass**)  F(3, 4164) = 136, P < 0.001 | Present | SSP1 | 0.3 | 0.3, 0.4 | **< 0.001** |
|  | Present | SSP2 | 0.1 | 0.07, 0.2 | **< 0.001** |
|  | Present | SSP5 | -0.08 | -0.13, -0.02 | **0.002** |
|  | SSP1 | SSP2 | -0.2 | -0.3, -0.1 | **< 0.001** |
|  | SSP1 | SSP5 | -0.4 | -0.4, -0.3 | **< 0.001** |
|  | SSP2 | SSP5 | -0.2 | -0.2, -0.1 | **< 0.001** |
| **Scenario** (when solar farms are managed with **meadow margins**)  F(3, 4164) = 12.5, P < 0.001 | Present | SSP1 | 0.3 | 0.1, 0.4 | **< 0.001** |
|  | Present | SSP2 | 0.13 | -0.02, 0.29 | 0.12 |
|  | Present | SSP5 | -0.08 | -0.23, 0.08 | 0.58 |
|  | SSP1 | SSP2 | -0.14 | -0.23, 0.02 | 0.12 |
|  | SSP1 | SSP5 | -0.3 | -0.5, -0.2 | **< 0.001** |
|  | SSP2 | SSP5 | -0.21 | -0.37, -0.05 | **0.002** |
| **Management**  F(1, 8334) = 24,815, P < 0.001 | Turf grass | Meadow margins | 3.6 | 3.6, 3.7 | **< 0.001** |

*Bumblebee foraging and nesting resources across land use scenarios*

**Text S7:** *Bumblebee foraging and nesting resources across land use scenarios*

At the landscape scale, total bumblebee foraging and nesting resources were greatest in *Sustainability*, followed by *Middle of the Road* and the present day and *Fossil-fuelled Development* (Figure S2a and b, Tables S11 and 12). There were no significant differences in total resources at the landscape scale between the present day and the least sustainable future land use scenario, *Fossil-fuelled Development,* and in terms of nesting resources, totals were not significantly different between *Sustainability* and *Middle of the Road* (Figure S2a and b, Tables S11 and 12). The differences between scenarios at the solar farm foraging zone scale mirrored those of the wider landscape, in terms of both total foraging and nesting resources (Figure S2c and d, Tables S13 and 14).


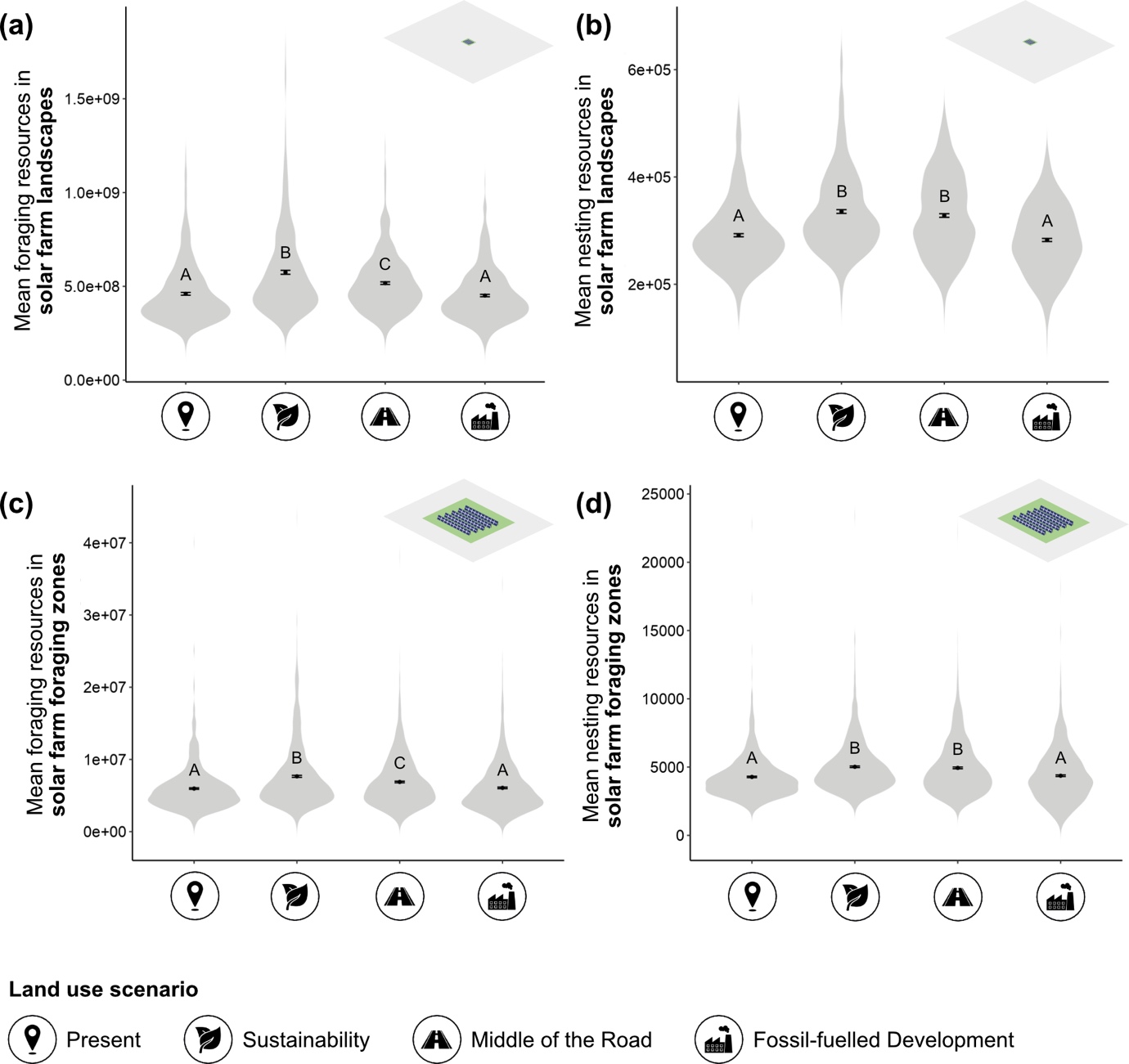


**Figure S2.** Distributions of the mean (a) foraging resources present in solar farm landscapes (*n* = 473), (b) nesting resources present in solar farm landscapes (*n* = 473), (c) foraging resources present in solar farm foraging zones (*n* = 1,042) and (d) nesting resources present in solar farm foraging zones (*n* = 1,042) across land use scenarios. Landscapes are 10 km squares centred on each solar farm and foraging zones extend 0 – 500 m from each solar farm boundary. Black points show the sample-level mean and error bars represent the standard error on this sample-level mean. Within each plot, points that share letters are not significantly different at the P < 0.05 level according to ANOVA and Tukey post-hoc analyses. Data were transformed before analysis using Box-Cox methods to meet statistical test assumptions in most cases. Land use scenario icons were reproduced from the Noun Project (<https://thenounproject.com>).

**Table S11.** Analysis of variance (ANOVA) and post-hoc Tukey analyses results evaluating differences in total floral resources in 10 km landscapes surrounding solar farms managed as turf grass (*n* = 473) under different land use scenarios where “SSP1” refers to *Sustainability*, “SSP2” to *Middle of the Road* and “SSP5” to *Fossil-fuelled Development*. ANOVA results are displayed under the effect name. “Group 1” and “Group 2” refer to the treatment groups being compared in Tukey analyses and “CI” refers to “confidence intervals”. Bold values indicate a significant difference at the P < 0.05 level between groups. Total floral resource values were transformed using Box-Cox methods, where λ = -0.59, to meet test assumptions of normality.

| **Effect** | **Group 1** | **Group 2** | **Estimate** | **CI** | **P** |
| --- | --- | --- | --- | --- | --- |
| **Scenario** (when solar farms are managed as **turf grass**)  F(3, 1888) = 58.9, P < 0.001 | Present | SSP1 | 0.000002 | 0.000001, 0.000002 | **< 0.001** |
|  | Present | SSP2 | 0.000001 | 0.0000007, 0.000002 | **< 0.001** |
|  | Present | SSP5 | -0.00000006 | -0.0000005, 0.0000003 | 0.98 |
|  | SSP1 | SSP2 | -0.0000006 | -0.000001, -0.0000001 | **0.003** |
|  | SSP1 | SSP5 | -0.000002 | -0.000002, -0.000001 | **< 0.001** |
|  | SSP2 | SSP5 | -0.000001 | -0.000002, -0.000008 | **< 0.001** |

**Table S12.** Analysis of variance (ANOVA) and post-hoc Tukey analyses results evaluating differences in total nesting resources in 10 km landscapes surrounding solar farms managed as turf grass (*n* = 473) under different land use scenarios where “SSP1” refers to *Sustainability*, “SSP2” to *Middle of the Road* and “SSP5” to *Fossil-fuelled Development*. ANOVA results are displayed under the effect name. “Group 1” and “Group 2” refer to the treatment groups being compared in Tukey analyses and “CI” refers to “confidence intervals”. Bold values indicate a significant difference at the P < 0.05 level between groups. Total nesting resource values had a normal distribution and therefore did not require transformation to meet test assumptions.

| **Effect** | **Group 1** | **Group 2** | **Estimate** | **CI** | **P** |
| --- | --- | --- | --- | --- | --- |
| **Scenario** (when solar farms are managed as **turf grass**)  F(3, 1888) = 70.0, P < 0.001 | Present | SSP1 | 44144 | 32705, 55584 | **< 0.001** |
|  | Present | SSP2 | 36722 | 25283, 48161 | **< 0.001** |
|  | Present | SSP5 | -8842 | -20281, 2597 | 0.19 |
|  | SSP1 | SSP2 | -7423 | -18862, 4016 | 0.34 |
|  | SSP1 | SSP5 | -52986 | -64425, -41547 | **< 0.001** |
|  | SSP2 | SSP5 | -45564 | -57003, 34125 | **< 0.001** |

**Table S13.** Analysis of variance (ANOVA) and post-hoc Tukey analyses results evaluating differences in total floral resources in in 0 – 500 m foraging zones surrounding solar farms (*n* = 1,042) under different land use scenarios where “SSP1” refers to *Sustainability*, “SSP2” to *Middle of the Road* and “SSP5” to *Fossil-fuelled Development*. ANOVA results are displayed under the effect name. “Group 1” and “Group 2” refer to the treatment groups being compared in Tukey analyses and “CI” refers to “confidence intervals”. Bold values indicate a significant difference at the P < 0.05 level between groups. Total floral resource values were transformed using Box-Cox methods, where λ = -0.02, to meet test assumptions of normality.

| **Effect** | **Group 1** | **Group 2** | **Estimate** | **CI** | **P** |
| --- | --- | --- | --- | --- | --- |
| **Scenario**  F(3, 4164) = 55.1, P < 0.001 | Present | SSP1 | 0.2 | 0.1, 0.2 | **< 0.001** |
|  | Present | SSP2 | 0.100 | 0.06, 0.10 | **< 0.001** |
|  | Present | SSP5 | -0.01 | -0.05, 0.03 | 0.91 |
|  | SSP1 | SSP2 | -0.06 | -0.10, -0.02 | **< 0.001** |
|  | SSP1 | SSP5 | -0.2 | -0.2, -0.1 | **< 0.001** |
|  | SSP2 | SSP5 | -0.11 | -0.15, -0.07 | **< 0.001** |

**Table S14.** Analysis of variance (ANOVA) and post-hoc Tukey analyses results evaluating differences in total nesting resources in in 0 – 500 m foraging zones surrounding solar farms (*n* = 1,042) under different land use scenarios where “SSP1” refers to *Sustainability*, “SSP2” to *Middle of the Road* and “SSP5” to *Fossil-fuelled Development*. ANOVA results are displayed under the effect name. “Group 1” and “Group 2” refer to the treatment groups being compared in Tukey analyses and “CI” refers to “confidence intervals”. Bold values indicate a significant difference at the P < 0.05 level between groups. Total nesting resource values were transformed using Box-Cox methods, where λ = -0.02, to meet test assumptions of normality.

| **Effect** | **Group 1** | **Group 2** | **Estimate** | **CI** | **P** |
| --- | --- | --- | --- | --- | --- |
| **Scenario**  F(3, 4164) = 55.1, P < 0.001 | Present | SSP1 | 0.2 | 0.1, 0.2 | **< 0.001** |
|  | Present | SSP2 | 0.2 | 0.1, 0.2 | **< 0.001** |
|  | Present | SSP5 | -0.02 | -0.07, 0.03 | 0.58 |
|  | SSP1 | SSP2 | -0.03 | -0.08, 0.01 | 0.27 |
|  | SSP1 | SSP5 | -0.2 | -0.3, -0.2 | **< 0.001** |
|  | SSP2 | SSP5 | -0.2 | -0.2, -0.1 | **< 0.001** |

*Drivers of change in foraging bumblebee density*

**Table S15**. Generalised linear model output describing the variation in the change in foraging bumblebee density in 10 km landscapes surrounding solar farms between the present day and (A) SSP1 (*Sustainability*), (B) SSP2 (*Middle of the Road*) and (C) SSP5 (*Fossil-fuelled Development*). Landcover variables represent the change in area of that landcover (ha), from the present day to the future. Landcover changes are at the landscape level, where the solar farm in the centre was managed as turf grass. Bold values indicate the variable is a significant predictor at the P < 0.05 level.

| 1. **Change in foraging bumblebee density from the present to SSP1** | | | | | | |
| --- | --- | --- | --- | --- | --- | --- |
| **Variable** | **β** | **SE** | **t** | | **P** | |
| Intercept | 0.011 | 0.002 | 4.50 | | **< 0.01** | |
| Agroforestry | -0.00002 | 0.00001 | -1.95 | | 0.05 | |
| Arable field margin | -0.3 | 0.2 | -1.66 | | 0.10 | |
| Cereal | -0.18 | 0.03 | -5.80 | | **< 0.01** | |
| Field beans | -0.00001 | 0.00001 | -1.14 | | 0.26 | |
| Flower patches | 0.5 | 0.4 | 1.38 | | 0.17 | |
| Grass ley | 0.162 | 0.008 | 19.89 | | **< 0.01** | |
| Hedgerow | -0.6 | 0.2 | -3.09 | | **< 0.01** | |
| Improved permanent grassland | -0.17 | 0.03 | -5.87 | | **< 0.01** | |
| Oilseed rape | -0.19 | 0.04 | -4.57 | | **< 0.01** | |
| Unimproved permanent grassland | 0.05 | 0.03 | 1.66 | | 0.10 | |
| Urban | 0.28 | 0.05 | 5.11 | | **< 0.01** | |
| Woodland | -0.000003 | 0.000003 | -1.04 | | 0.30 | |
| **Null deviance:** 0.18 on 472 degrees of freedom **AIC:** -3,022 | | | | | | |
| **Residual deviance:** 0.04 on 460 degrees of freedom | | | | | | |
| **(B) Change in foraging bumblebee density from the present to SSP2** | | | | | | |
| **Variable** | **β** | **SE** | **t** | | **P** | |
| Intercept | -0.0013 | 0.0007 | -1.77 | | 0.08 | |
| Agroforestry | 0.000012 | 0.000003 | 3.64 | | **< 0.01** | |
| Arable field margin | -0.00001 | 0.00004 | -0.24 | | 0.81 | |
| Cereal | -0.0000042 | 0.0000008 | -5.10 | | **< 0.01** | |
| Field beans | 0.00000 | 0.00001 | 0.06 | | 0.95 | |
| Flower patches | 0.0001 | 0.0002 | 0.28 | | 0.78 | |
| Grass ley | 0.0000002 | 0.0000004 | 0.34 | | 0.73 | |
| Hedgerow | 0.00016 | 0.00008 | 1.95 | | 0.05 | |
| Improved permanent grassland | -11 | 28 | -0.40 | | 0.69 | |
| Oilseed rape | -0.000001 | 0.000002 | -0.77 | | 0.44 | |
| Unimproved permanent grassland | 0.0000199 | 0.0000007 | 27.06 | | **< 0.01** | |
| Urban | 0.000010 | 0.000002 | 4.25 | | **< 0.01** | |
| Woodland | 0.0000158 | 0.0000004 | 38.89 | | **< 0.01** | |
| **Null deviance:** 0.27 on 472 degrees of freedom **AIC:** -3,519 | | | | | | |
| **Residual deviance:** 0.015 on 460 degrees of freedom | | | | | | |
| **(C) Change in foraging bumblebee density from the present to SSP5** | | | | | | |
| **Variable** | **β** | **SE** | | **t** | | **P** |
| Intercept | 0.001 | 0.001 | | 0.73 | | 0.47 |
| Agroforestry | 0.000018 | 0.000009 | | 2.12 | | **0.03** |
| Arable field margin | -0.00012 | 0.00005 | | -2.34 | | **0.02** |
| Cereal | 0.000009 | 0.000008 | | 1.04 | | 0.30 |
| Field beans | 0.00002 | 0.00001 | | 1.65 | | 0.10 |
| Flower patches | 0.00010 | 0.00022 | | 0.45 | | 0.65 |
| Grass ley | -0.000013 | 0.000001 | | -25.38 | | **< 0.01** |
| Hedgerow | -0.00021 | 0.00007 | | -3.11 | | **< 0.01** |
| Improved permanent grassland | 0.000010 | 0.000009 | | 1.21 | | 0.23 |
| Oilseed rape | 0.000008 | 0.000008 | | 1.00 | | 0.32 |
| Unimproved permanent grassland | 0.000030 | 0.000009 | | 3.51 | | **< 0.01** |
| Urban | 0.000005 | 0.000009 | | 0.56 | | 0.58 |
| Woodland | 0.000026 | 0.000008 | | 3.08 | | **< 0.01** |
| **Null deviance:** 0.24 on 472 degrees of freedom **AIC:** -3,333 | | | | | | |
| **Residual deviance:** 0.02 on 460 degrees of freedom | | | | | | |

**Table S16**. Generalised linear model output describing the variation in the change in foraging bumblebee density in 0- 500 m foraging zones surrounding solar farms between the present day and (A) SSP1 (*Sustainability*), (B) SSP2 (*Middle of the Road*) and (C) SSP5 (*Fossil-fuelled Development*). Landcover variables represent the change in area of that landcover (ha), from the present day to the future. Landcover changes are at the landscape and foraging zone level, where the solar farm in the centre was managed as turf grass. Bold values indicate the variable is a significant predictor at the P < 0.05 level.

|  | | 1. **Change in foraging bumblebee density from the present to SSP1** | | | | | | | |
| --- | --- | --- | --- | --- | --- | --- | --- | --- | --- |
|  | **Variable** | | **β** | **SE** | **t** | | **P** | |  |
| Foraging zone-level changes | Intercept | | 2.4 | 0.5 | 5.10 | | **< 0.01** | |  |
|  | Agroforestry | | -0.03 | 0.03 | -1.03 | | 0.30 | |  |
|  | Arable field margin | | 0.10 | 0.08 | 1.36 | | 0.17 | |  |
|  | Cereal | | -0.10 | 0.01 | -9.38 | | **< 0.01** | |  |
|  | Field beans | | -0.05 | 0.02 | -2.00 | | 0.05 | |  |
|  | Flower patches | | -0.1 | 0.1 | -0.56 | | 0.58 | |  |
|  | Grass ley | | 0.05 | 0.01 | 4.41 | | **< 0.01** | |  |
|  | Hedgerow | | -0.46 | 0.09 | -4.87 | | **< 0.01** | |  |
|  | Improved permanent grassland | | -0.09 | 0.01 | -9.03 | | **< 0.01** | |  |
|  | Oilseed rape | | -0.03 | 0.01 | -2.23 | | **0.03** | |  |
|  | Unimproved permanent grassland | | -0.02 | 0.02 | -1.57 | | 0.12 | |  |
|  | Urban | | 0.11 | 0.01 | 8.12 | | **< 0.01** | |  |
|  | Woodland | | -0.06 | 0.01 | -4.89 | | **< 0.01** | |  |
| Landscape-level changes | Agroforestry | | -0.003 | 0.002 | -1.16 | | 0.25 | |  |
|  | Arable field margin | | 0.006 | 0.004 | 1.42 | | 0.16 | |  |
|  | Cereal | | -0.0001 | 0.0006 | -0.21 | | 0.84 | |  |
|  | Field beans | | -0.000 | 0.001 | -0.07 | | 0.94 | |  |
|  | Flower patches | | -0.019 | 0.008 | -2.31 | | **0.02** | |  |
|  | Grass ley | | 0.0004 | 0.0002 | 2.62 | | **0.01** | |  |
|  | Hedgerow | | -0.008 | 0.004 | -2.04 | | **0.04** | |  |
|  | Improved permanent grassland | | 0.0003 | 0.0006 | 0.44 | | 0.66 | |  |
|  | Oilseed rape | | -0.0007 | 0.0008 | -0.81 | | 0.42 | |  |
|  | Unimproved permanent grassland | | 0.0011 | 0.0006 | 1.68 | | 0.09 | |  |
|  | Urban | | 0.002 | 0.001 | 1.73 | | 0.08 | |  |
|  | Woodland | | 0.0007 | 0.0007 | 0.99 | | 0.32 | |  |
|  | **Null deviance:** 17,367 on 1,041 degrees of freedom **AIC:** -3,022 | | | | | | | |  |
|  | **Residual deviance:** 7,580 on 1017 degrees of freedom | | | | | | | |  |
|  | **(B) Change in foraging bumblebee density from the present to SSP2** | | | | | | | |  |
|  | **Variable** | | **β** | **SE** | **t** | | **P** | |  |
| Foraging zone-level changes | Intercept | | -0.2 | 0.2 | -0.90 | | 0.37 | |  |
|  | Agroforestry | | 0.00 | 0.04 | -0.10 | | 0.92 | |  |
|  | Arable field margin | | 0.2 | 0.2 | 1.18 | | 0.24 | |  |
|  | Cereal | | 0.00 | 0.04 | 0.05 | | 0.96 | |  |
|  | Field beans | | 0.06 | 0.05 | 1.31 | | 0.19 | |  |
|  | Flower patches | | -0.5 | 0.5 | -0.92 | | 0.36 | |  |
|  | Grass ley | | -0.01 | 0.01 | -1.05 | | 0.29 | |  |
|  | Hedgerow | | 0.2 | 0.1 | 1.68 | | 0.09 | |  |
|  | Improved permanent grassland | | 0.01 | 0.04 | 0.28 | | 0.78 | |  |
|  | Oilseed rape | | 0.06 | 0.04 | 1.67 | | 0.10 | |  |
|  | Unimproved permanent grassland | | 0.10 | 0.04 | 2.55 | | **0.01** | |  |
|  | Urban | | 0.05 | 0.04 | 1.24 | | 0.21 | |  |
|  | Woodland | | 0.07 | 0.04 | 1.90 | | 0.06 | |  |
| Landscape-level changes | Agroforestry | | 0.003 | 0.003 | 1.06 | | 0.29 | |  |
|  | Arable field margin | | 0.02 | 0.01 | 1.35 | | 0.18 | |  |
|  | Cereal | | 0.001 | 0.002 | 0.31 | | 0.76 | |  |
|  | Field beans | | 0.004 | 0.005 | 0.76 | | 0.45 | |  |
|  | Flower patches | | -0.09 | 0.08 | -1.20 | | 0.23 | |  |
|  | Grass ley | | 0.0000 | 0.0001 | -0.02 | | 0.99 | |  |
|  | Hedgerow | | 0.03 | 0.02 | 1.33 | | 0.18 | |  |
|  | Improved permanent grassland | | 0.001 | 0.002 | 0.61 | | 0.54 | |  |
|  | Oilseed rape | | 0.000 | 0.002 | 0.12 | | 0.90 | |  |
|  | Unimproved permanent grassland | | 0.002 | 0.002 | 0.89 | | 0.37 | |  |
|  | Urban | | 0.003 | 0.002 | 1.37 | | 0.17 | |  |
|  | Woodland | | 0.002 | 0.002 | 0.82 | | 0.41 | |  |
|  | **Null deviance:** 13,638 on 1,041 degrees of freedom **AIC:** 4,932 | | | | | | | |  |
|  | **Residual deviance:** 6,598 on 1,017 degrees of freedom | | | | | | | |  |
|  | **(C) Change in foraging bumblebee density from the present to SSP5** | | | | | | | |  |
|  | **Variable** | | **β** | **SE** | | **t** | | **P** |  |
| Foraging zone-level changes | Intercept | | -0.1 | 0.3 | | -0.17 | | 0.87 |  |
|  | Agroforestry | | 0.000 | 0.002 | | 0.19 | | 0.85 |  |
|  | Arable field margin | | -0.02 | 0.01 | | -1.75 | | 0.08 |  |
|  | Cereal | | 0.000 | 0.002 | | 0.20 | | 0.84 |  |
|  | Field beans | | 0.001 | 0.003 | | 0.18 | | 0.86 |  |
|  | Flower patches | | 0.03 | 0.05 | | 0.53 | | 0.60 |  |
|  | Grass ley | | -0.0005 | 0.0001 | | -3.99 | | **< 0.01** |  |
|  | Hedgerow | | -0.02 | 0.02 | | -1.25 | | 0.21 |  |
|  | Improved permanent grassland | | 0.000 | 0.002 | | 0.01 | | 0.99 |  |
|  | Oilseed rape | | -0.001 | 0.002 | | -0.62 | | 0.54 |  |
|  | Unimproved permanent grassland | | 0.001 | 0.002 | | 0.33 | | 0.74 |  |
|  | Urban | | 0.000 | 0.002 | | 0.02 | | 0.98 |  |
|  | Woodland | | 0.001 | 0.002 | | 0.42 | | 0.67 |  |
| Landscape-level changes | Agroforestry | | 0.000 | 0.002 | | 0.19 | | 0.85 |  |
|  | Arable field margin | | -0.02 | 0.01 | | -1.75 | | 0.08 |  |
|  | Cereal | | 0.000 | 0.002 | | 0.20 | | 0.84 |  |
|  | Field beans | | 0.001 | 0.003 | | 0.18 | | 0.86 |  |
|  | Flower patches | | 0.03 | 0.05 | | 0.53 | | 0.60 |  |
|  | Grass ley | | -0.0005 | 0.0001 | | -3.99 | | **< 0.01** |  |
|  | Hedgerow | | -0.02 | 0.02 | | -1.25 | | 0.21 |  |
|  | Improved permanent grassland | | 0.000 | 0.002 | | 0.01 | | 0.99 |  |
|  | Oilseed rape | | -0.001 | 0.002 | | -0.62 | | 0.54 |  |
|  | Unimproved permanent grassland | | 0.001 | 0.002 | | 0.33 | | 0.74 |  |
|  | Urban | | 0.000 | 0.002 | | 0.02 | | 0.98 |  |
|  | Woodland | | 0.001 | 0.002 | | 0.42 | | 0.67 |  |
|  | **Null deviance:** 13,247 on 1,041 degrees of freedom **AIC:** 4,814 | | | | | | | |  |
|  | **Residual deviance:** 5,890 on 1,017 degrees of freedom | | | | | | | |  |

**Table S17.** Generalised linear model output describing the variation in the change in foraging bumblebee density inside solar farms between the present day and (A) SSP1 (*Sustainability*), (B) SSP2 (*Middle of the Road*) and (C) SSP5 (*Fossil-fuelled Development*). Landcover variables represent the change in area of that landcover (ha), from the present day to the future. Landcover changes are at the landscape and foraging zone level, where the solar farm in the centre was managed as turf grass. Bold values indicate the variable is a significant predictor at the P < 0.05 level.

|  | | 1. **Change in foraging bumblebee density from the present to SSP1** | | | | | | | |
| --- | --- | --- | --- | --- | --- | --- | --- | --- | --- |
|  | **Variable** | | **β** | **SE** | **t** | | **P** | |  |
| Foraging zone-level changes | Intercept | | 0.10 | 0.07 | 1.32 | | 0.19 | |  |
|  | Agroforestry | | 0.006 | 0.005 | 1.20 | | 0.23 | |  |
|  | Arable field margin | | 0.04 | 0.01 | 2.98 | | **< 0.01** | |  |
|  | Cereal | | -0.012 | 0.002 | -6.88 | | **< 0.01** | |  |
|  | Field beans | | -0.014 | 0.004 | -3.80 | | **< 0.01** | |  |
|  | Flower patches | | -0.00 | 0.02 | -0.19 | | 0.85 | |  |
|  | Grass ley | | -0.004 | 0.002 | -2.32 | | 0.02 | |  |
|  | Hedgerow | | 0.02 | 0.02 | 1.36 | | 0.18 | |  |
|  | Improved permanent grassland | | -0.005 | 0.002 | -3.05 | | **< 0.01** | |  |
|  | Oilseed rape | | -0.016 | 0.002 | -8.72 | | **< 0.01** | |  |
|  | Unimproved permanent grassland | | 0.002 | 0.002 | 0.82 | | 0.41 | |  |
|  | Urban | | 0.005 | 0.002 | 2.47 | | **0.01** | |  |
|  | Woodland | | 0.003 | 0.002 | 1.31 | | 0.19 | |  |
| Landscape-level changes | Agroforestry | | -0.0002 | 0.0003 | -0.67 | | 0.50 | |  |
|  | Arable field margin | | -0.0012 | 0.0006 | -1.91 | | 0.06 | |  |
|  | Cereal | | -0.0003 | 0.0001 | -3.29 | | **< 0.01** | |  |
|  | Field beans | | 0.0001 | 0.0002 | 0.28 | | 0.78 | |  |
|  | Flower patches | | 0.003 | 0.001 | 1.93 | | 0.05 | |  |
|  | Grass ley | | 0.00001 | 0.00003 | -0.52 | | 0.60 | |  |
|  | Hedgerow | | 0.0007 | 0.0006 | 1.11 | | 0.27 | |  |
|  | Improved permanent grassland | | -0.0002 | 0.0001 | -2.65 | | **0.01** | |  |
|  | Oilseed rape | | -0.0006 | 0.0001 | -4.82 | | **< 0.01** | |  |
|  | Unimproved permanent grassland | | -0.0001 | 0.0001 | -0.90 | | 0.37 | |  |
|  | Urban | | 0.0006 | 0.0002 | 3.54 | | **< 0.01** | |  |
|  | Woodland | | -0.0001 | 0.0001 | -0.48 | | 0.63 | |  |
|  | **Null deviance:** 715 on 1,041 degrees of freedom **AIC:** 1,234 | | | | | | | |  |
|  | **Residual deviance:** 190 on 1,017 degrees of freedom | | | | | | | |  |
|  | **(B) Change in foraging bumblebee density from the present to SSP2** | | | | | | | |  |
|  | **Variable** | | **β** | **SE** | **t** | | **P** | |  |
| Foraging zone-level changes | Intercept | | 0.03 | 0.04 | 0.67 | | 0.50 | |  |
|  | Agroforestry | | -0.009 | 0.007 | -1.32 | | 0.19 | |  |
|  | Arable field margin | | -0.09 | 0.03 | -2.50 | | **0.01** | |  |
|  | Cereal | | -0.013 | 0.006 | -2.14 | | **0.03** | |  |
|  | Field beans | | -0.001 | 0.008 | -0.16 | | 0.87 | |  |
|  | Flower patches | | 0.10 | 0.09 | 1.14 | | 0.25 | |  |
|  | Grass ley | | -0.004 | 0.002 | -2.46 | | **0.01** | |  |
|  | Hedgerow | | 0.07 | 0.02 | 2.87 | | **< 0.01** | |  |
|  | Improved permanent grassland | | -0.008 | 0.006 | -1.25 | | 0.21 | |  |
|  | Oilseed rape | | -0.016 | 0.006 | -2.70 | | **0.01** | |  |
|  | Unimproved permanent grassland | | 0.000 | 0.006 | 0.05 | | 0.96 | |  |
|  | Urban | | -0.004 | 0.006 | -0.61 | | 0.54 | |  |
|  | Woodland | | 0.000 | 0.006 | 0.04 | | 0.97 | |  |
| Landscape-level changes | Agroforestry | | -0.0004 | 0.0005 | -0.81 | | 0.42 | |  |
|  | Arable field margin | | -0.014 | 0.002 | -6.09 | | **< 0.01** | |  |
|  | Cereal | | -0.0003 | 0.0004 | -0.90 | | 0.37 | |  |
|  | Field beans | | 0.0004 | 0.0008 | 0.42 | | 0.67 | |  |
|  | Flower patches | | 0.04 | 0.01 | 3.20 | | **< 0.01** | |  |
|  | Grass ley | | 0.00002 | 0.00002 | 0.66 | | 0.51 | |  |
|  | Hedgerow | | 0.003 | 0.004 | 0.63 | | 0.53 | |  |
|  | Improved permanent grassland | | -0.0004 | 0.0004 | -1.02 | | 0.31 | |  |
|  | Oilseed rape | | -0.0006 | 0.0004 | -1.50 | | 0.13 | |  |
|  | Unimproved permanent grassland | | -0.0002 | 0.0004 | -0.54 | | 0.59 | |  |
|  | Urban | | -0.0003 | 0.0004 | -0.74 | | 0.46 | |  |
|  | Woodland | | -0.0002 | 0.0004 | -0.52 | | 0.61 | |  |
|  | **Null deviance:** 1,183 on 1,041 degrees of freedom **AIC:** 1,211 | | | | | | | |  |
|  | **Residual deviance:** 186 on 1,017 degrees of freedom | | | | | | | |  |
|  | **(C) Change in foraging bumblebee density from the present to SSP5** | | | | | | | |  |
|  | **Variable** | | **β** | **SE** | | **t** | | **P** |  |
| Foraging zone-level changes | Intercept | | 0.03 | 0.06 | | 0.43 | | 0.67 |  |
|  | Agroforestry | | 0.004 | 0.004 | | 0.86 | | 0.39 |  |
|  | Arable field margin | | -0.03 | 0.03 | | -1.22 | | 0.22 |  |
|  | Cereal | | -0.007 | 0.004 | | -1.62 | | 0.11 |  |
|  | Field beans | | -0.004 | 0.006 | | -0.69 | | 0.49 |  |
|  | Flower patches | | 0.08 | 0.07 | | 1.11 | | 0.27 |  |
|  | Grass ley | | -0.008 | 0.002 | | -4.23 | | **< 0.01** |  |
|  | Hedgerow | | 0.12 | 0.03 | | 4.09 | | **< 0.01** |  |
|  | Improved permanent grassland | | 0.000 | 0.004 | | -0.08 | | 0.94 |  |
|  | Oilseed rape | | -0.012 | 0.004 | | -2.88 | | **< 0.01** |  |
|  | Unimproved permanent grassland | | 0.008 | 0.004 | | 1.86 | | 0.06 |  |
|  | Urban | | -0.005 | 0.004 | | -1.23 | | 0.22 |  |
|  | Woodland | | 0.009 | 0.004 | | 2.07 | | **0.04** |  |
| Landscape-level changes | Agroforestry | | -0.0000 | 0.0004 | | -0.09 | | 0.92 |  |
|  | Arable field margin | | -0.022 | 0.002 | | -8.82 | | **< 0.01** |  |
|  | Cereal | | 0.0002 | 0.0004 | | 0.40 | | 0.69 |  |
|  | Field beans | | 0.0004 | 0.0006 | | 0.65 | | 0.52 |  |
|  | Flower patches | | 0.05 | 0.01 | | 5.00 | | **< 0.01** |  |
|  | Grass ley | | -0.00011 | 0.00002 | | -4.63 | | **< 0.01** |  |
|  | Hedgerow | | -0.007 | 0.003 | | -2.06 | | **0.04** |  |
|  | Improved permanent grassland | | -0.0001 | 0.0004 | | -0.25 | | 0.80 |  |
|  | Oilseed rape | | -0.0003 | 0.0004 | | -0.68 | | 0.50 |  |
|  | Unimproved permanent grassland | | 0.0001 | 0.0004 | | 0.21 | | 0.83 |  |
|  | Urban | | -0.0002 | 0.0004 | | -0.36 | | 0.72 |  |
|  | Woodland | | 0.0001 | 0.0004 | | 0.28 | | 0.78 |  |
|  | **Null deviance:** 1,140 on 1,041 degrees of freedom **AIC:** 1,406 | | | | | | | |  |
|  | **Residual deviance:** 224 on 1,017 degrees of freedom | | | | | | | |  |

**Table S18.** Generalised linear model output describing the variation in the change in foraging bumblebee density inside solar farms between the present day and (A) SSP1 (*Sustainability*), (B) SSP2 (*Middle of the Road*) and (C) SSP5 (*Fossil-fuelled Development*). Landcover variables represent the change in area of that landcover (ha), from the present day to the future. Landcover changes are at the landscape and foraging zone level, where the solar farm in the centre was managed with meadow margins. Bold values indicate the variable is a significant predictor at the P < 0.05 level.

|  | | 1. **Change in foraging bumblebee density from the present to SSP1** | | | | | | | |
| --- | --- | --- | --- | --- | --- | --- | --- | --- | --- |
|  | **Variable** | | **β** | **SE** | **t** | | **P** | |  |
| Foraging zone-level changes | Intercept | | 0.1 | 0.2 | 0.69 | | 0.49 | |  |
|  | Agroforestry | | 0.00 | 0.01 | 0.20 | | 0.84 | |  |
|  | Arable field margin | | 0.04 | 0.03 | 1.56 | | 0.12 | |  |
|  | Cereal | | -0.030 | 0.004 | -8.30 | | **< 0.01** | |  |
|  | Field beans | | -0.034 | 0.008 | -4.32 | | **< 0.01** | |  |
|  | Flower patches | | 0.01 | 0.04 | 0.30 | | 0.76 | |  |
|  | Grass ley | | -0.013 | 0.003 | -3.82 | | **< 0.01** | |  |
|  | Hedgerow | | -0.03 | 0.03 | -0.96 | | 0.33 | |  |
|  | Improved permanent grassland | | -0.014 | 0.003 | -4.16 | | **< 0.01** | |  |
|  | Oilseed rape | | -0.040 | 0.004 | -10.37 | | **< 0.01** | |  |
|  | Unimproved permanent grassland | | 0.000 | 0.005 | -0.01 | | 1.00 | |  |
|  | Urban | | 0.003 | 0.004 | 0.69 | | 0.49 | |  |
|  | Woodland | | 0.001 | 0.004 | 0.33 | | 0.74 | |  |
| Landscape-level changes | Agroforestry | | 0.0003 | 0.0007 | 0.39 | | 0.70 | |  |
|  | Arable field margin | | -0.004 | 0.001 | -3.13 | | **< 0.01** | |  |
|  | Cereal | | -0.0008 | 0.0002 | -3.46 | | **< 0.01** | |  |
|  | Field beans | | -0.0001 | 0.0004 | -0.25 | | 0.80 | |  |
|  | Flower patches | | 0.010 | 0.003 | 3.78 | | **< 0.01** | |  |
|  | Grass ley | | -0.00001 | 0.00005 | -0.15 | | 0.88 | |  |
|  | Hedgerow | | 0.002 | 0.001 | 1.20 | | 0.23 | |  |
|  | Improved permanent grassland | | -0.0006 | 0.0002 | -3.11 | | **< 0.01** | |  |
|  | Oilseed rape | | -0.0013 | 0.0003 | -5.01 | | **< 0.01** | |  |
|  | Unimproved permanent grassland | | -0.0002 | 0.0002 | -1.05 | | 0.29 | |  |
|  | Urban | | 0.0017 | 0.0003 | 4.88 | | **< 0.01** | |  |
|  | Woodland | | -0.0003 | 0.0002 | -1.24 | | 0.21 | |  |
|  | **Null deviance:** 3,543 on 1,041 degrees of freedom **AIC:** 2,784 | | | | | | | |  |
|  | **Residual deviance:** 839 on 1,017 degrees of freedom | | | | | | | |  |
|  | **(B) Change in foraging bumblebee density from the present to SSP2** | | | | | | | |  |
|  | **Variable** | | **β** | **SE** | **t** | | **P** | |  |
| Foraging zone-level changes | Intercept | | 0.06 | 0.09 | 0.69 | | 0.49 | |  |
|  | Agroforestry | | -0.02 | 0.02 | -1.22 | | 0.22 | |  |
|  | Arable field margin | | -0.19 | 0.08 | -2.38 | | **0.02** | |  |
|  | Cereal | | -0.03 | 0.01 | -2.05 | | **0.04** | |  |
|  | Field beans | | 0.00 | 0.02 | 0.02 | | 0.98 | |  |
|  | Flower patches | | 0.1 | 0.2 | 0.61 | | 0.54 | |  |
|  | Grass ley | | -0.011 | 0.004 | -3.05 | | **< 0.01** | |  |
|  | Hedgerow | | 0.07 | 0.06 | 1.33 | | 0.18 | |  |
|  | Improved permanent grassland | | -0.02 | 0.01 | -1.16 | | 0.25 | |  |
|  | Oilseed rape | | -0.04 | 0.01 | -2.59 | | **0.01** | |  |
|  | Unimproved permanent grassland | | 0.00 | 0.01 | 0.28 | | 0.78 | |  |
|  | Urban | | -0.01 | 0.01 | -0.60 | | 0.55 | |  |
|  | Woodland | | 0.00 | 0.01 | 0.01 | | 0.99 | |  |
| Landscape-level changes | Agroforestry | | -0.002 | 0.001 | -1.57 | | 0.12 | |  |
|  | Arable field margin | | -0.040 | 0.005 | -7.72 | | **< 0.01** | |  |
|  | Cereal | | -0.0012 | 0.0009 | -1.44 | | 0.15 | |  |
|  | Field beans | | 0.000 | 0.002 | 0.15 | | 0.88 | |  |
|  | Flower patches | | 0.11 | 0.03 | 3.78 | | **< 0.01** | |  |
|  | Grass ley | | 0.0000 | 0.0001 | 0.90 | | 0.37 | |  |
|  | Hedgerow | | 0.01 | 0.01 | 1.09 | | 0.27 | |  |
|  | Improved permanent grassland | | -0.0015 | 0.0009 | -1.69 | | 0.09 | |  |
|  | Oilseed rape | | -0.0015 | 0.0009 | -1.80 | | 0.07 | |  |
|  | Unimproved permanent grassland | | -0.0012 | 0.0009 | -1.33 | | 0.18 | |  |
|  | Urban | | -0.0010 | 0.0009 | -1.08 | | 0.28 | |  |
|  | Woodland | | -0.0011 | 0.0009 | -1.20 | | 0.23 | |  |
|  | **Null deviance:** 5,777 on 1,041 degrees of freedom **AIC:** 2,937 | | | | | | | |  |
|  | **Residual deviance:** 972 on 1,017 degrees of freedom | | | | | | | |  |
|  | **(C) Change in foraging bumblebee density from the present to SSP5** | | | | | | | |  |
|  | **Variable** | | **β** | **SE** | | **t** | | **P** |  |
| ffer-level changes | Intercept | | 0.0 | 0.1 | | 0.20 | | 0.84 |  |
|  | Agroforestry | | 0.01 | 0.01 | | 0.95 | | 0.34 |  |
|  | Arable field margin | | -0.12 | 0.06 | | -1.82 | | 0.07 |  |
|  | Cereal | | -0.013 | 0.009 | | -1.44 | | 0.15 |  |
|  | Field beans | | -0.01 | 0.01 | | -0.40 | | 0.69 |  |
|  | Flower patches | | 0.2 | 0.2 | | 1.09 | | 0.28 |  |
|  | Grass ley | | -0.019 | 0.004 | | -4.76 | | **< 0.01** |  |
|  | Hedgerow | | 0.16 | 0.07 | | 2.45 | | **0.01** |  |
|  | Improved permanent grassland | | 0.001 | 0.009 | | 0.07 | | 0.95 |  |
|  | Oilseed rape | | -0.024 | 0.009 | | -2.61 | | **0.01** |  |
|  | Unimproved permanent grassland | | 0.02 | 0.01 | | 1.97 | | 0.05 |  |
|  | Urban | | -0.01 | 0.01 | | -1.13 | | 0.26 |  |
|  | Woodland | | 0.020 | 0.009 | | 2.14 | | **0.03** |  |
| Landscape-level changes | Agroforestry | | -0.000 | 0.001 | | -0.40 | | 0.69 |  |
|  | Arable field margin | | -0.054 | 0.006 | | -9.72 | | **< 0.01** |  |
|  | Cereal | | 0.0001 | 0.0009 | | 0.09 | | 0.93 |  |
|  | Field beans | | 0.001 | 0.001 | | 0.45 | | 0.65 |  |
|  | Flower patches | | 0.14 | 0.02 | | 5.76 | | **< 0.01** |  |
|  | Grass ley | | -0.00022 | 0.00006 | | -3.97 | | **< 0.01** |  |
|  | Hedgerow | | -0.013 | 0.007 | | -1.85 | | 0.06 |  |
|  | Improved permanent grassland | | -0.0006 | 0.0009 | | -0.64 | | 0.52 |  |
|  | Oilseed rape | | -0.0008 | 0.0009 | | -0.84 | | 0.40 |  |
|  | Unimproved permanent grassland | | -0.0002 | 0.0009 | | -0.23 | | 0.82 |  |
|  | Urban | | -0.0007 | 0.0009 | | -0.74 | | 0.46 |  |
|  | Woodland | | -0.0001 | 0.0009 | | -0.14 | | 0.89 |  |
|  | **Null deviance:** 5,620 on 1,041 degrees of freedom **AIC:** 3,117 | | | | | | | |  |
|  | **Residual deviance:** 1,155 on 1,017 degrees of freedom | | | | | | | |  |

**
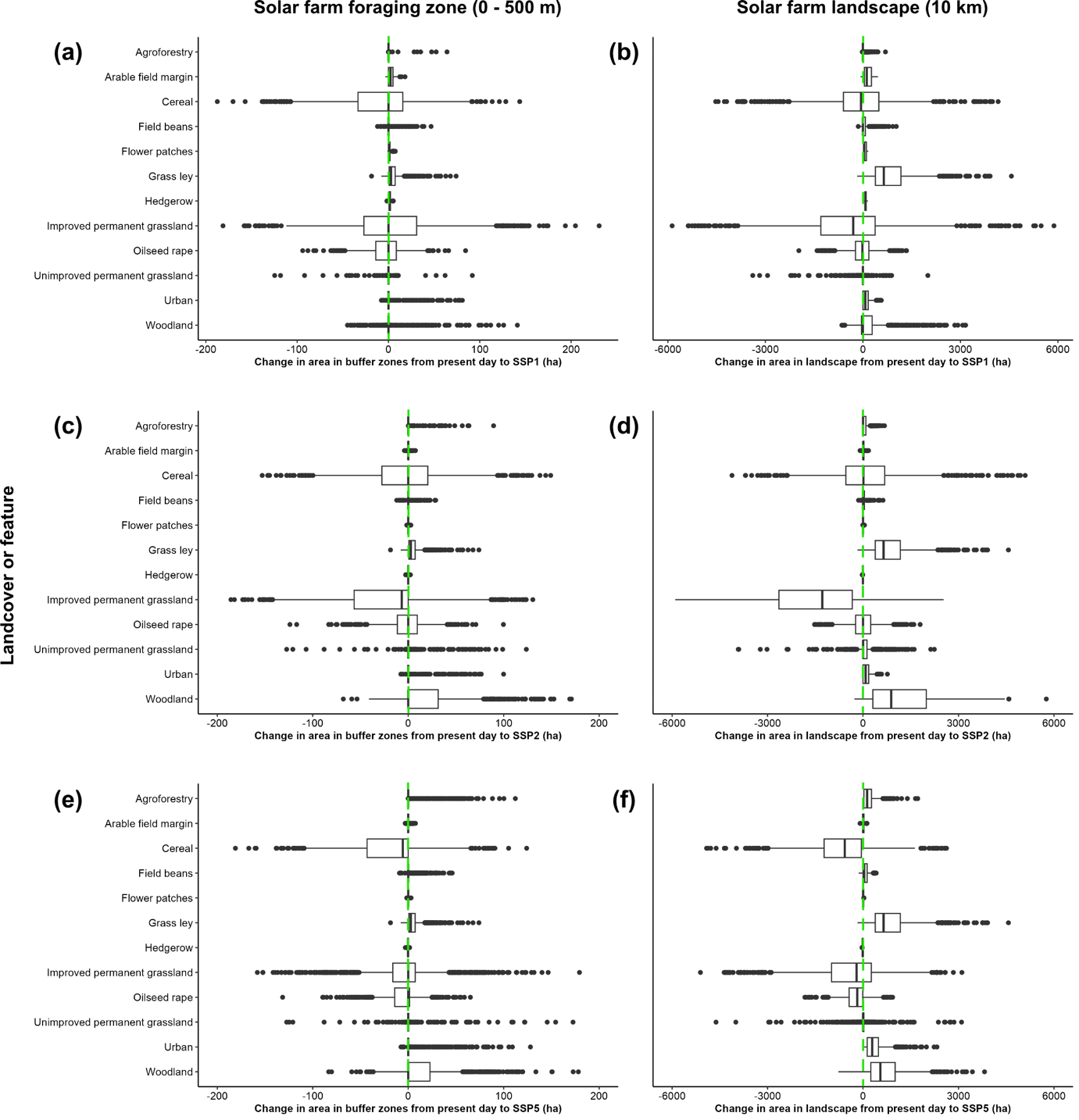
**

**Figure S3.** Box plots of landcover and feature area change included in generalised linear models to explain variation in foraging bumblebee density change from the present day to future scenarios where (a) and (b) show change from the present day to SSP1 (*Sustainability*), (c) and (d) to SSP2 (*Middle of the Road*) and (e) and (f) to SSP5 (*Fossil-fuelled Development*). Change in area (ha) is shown at two spatial scales, where (a), (c) and (e) show change inside 0 – 500 m foraging zones surrounding solar farms (*n* = 1,042; panes a, c and e) and (b), (d) and (f) show change in 10 km landscapes surrounding solar farms (*n* = 473). Dashed green lines at zero change in area are present to better distinguish positive or negative changes.

**References**

Baston, D. (2023) *exactextractr: Fast Extraction from Raster Datasets using Polygons*. Available at: <https://cran.r-project.org/web/packages/exactextractr/index.html>.

Blaydes, H., Gardner, E., Whyatt, D., Potts, S. G. and Armstrong, A. (2022) Solar park management and design to boost bumble bee populations. *Environmental Research Letters*, 17(4), 044002. <http://dx.doi.org/10.1088/1748-9326/ac5840>.

Brown, C., Seo, B., Alexander, P., Burton, V., Chacón‐Montalván, E., Dunford, R., Merkle, M., Harrison, P., Prestele, R. and Robinson, E. L. (2022) Agent‐based modeling of alternative futures in the British land use system. *Earth's Future,* 10(11), e2022EF002905. <https://doi.org/10.1029/2022EF002905>.

CCC (2018) Land use: Reducing emissions and preparing for climate change. Committee on Climate Change. Available at: <https://www.theccc.org.uk/wp-content/uploads/2018/11/Land-use-Reducing-emissions-and-preparing-for-climate-change-CCC-2018-1.pdf>.

Gardner, E., Breeze, T. D., Clough, Y., Smith, H. G., Baldock, K. C., Campbell, A., Garratt, M. P., Gillespie, M. A., Kunin, W. E., McKerchar, M., Memmott, J., Potts, S.G., Senapathi, D., Stone, G.N., Wackers, F., Westbury, D.B., Wilby, A. and Oliver, T.H. (2020) Reliably predicting pollinator abundance: Challenges of calibrating process‐based ecological models. *Methods in Ecology and Evolution,* 11(12), 1673-1689. <https://doi.org/10.1111/2041-210X.13483>.

Gardner, E., Breeze, T. D., Clough, Y., Smith, H. G., Baldock, K. C., Campbell, A., Garratt, M. P., Gillespie, M. A., Kunin, W. E., McKerchar, M., Potts, S.G., Senapathi, D., Stone, G.N., Wackers, F., Westbury, D.B., Wilby, A. and Oliver, T.H. (2021) Field boundary features can stabilise bee populations and the pollination of mass‐flowering crops in rotational systems. *Journal of Applied Ecology,* 58(10), 2287-2304. <https://doi.org/10.1111/1365-2664.13948>.

Hijmans, R.J (2023a) *raster: Geographic Data Analysis and Modeling*. Available at: <https://cran.r-project.org/web/packages/raster/index.html>.

Hijmans, R.J (2023b) *terra: Spatial Data Analysis*. Available at: <https://cran.r-project.org/web/packages/terra/index.html>.

Image, M., Gardner, E. and Breeze, T. D. (2023) Co-benefits from tree planting in a typical English agricultural landscape: Comparing the relative effectiveness of hedgerows, agroforestry and woodland creation for improving crop pollination services. *Land Use Policy,* 125, 106497. <https://doi.org/10.1016/j.agee.2021.107755>.

Image, M., Gardner, E., Clough, Y., Smith, H. G., Baldock, K. C., Campbell, A., Garratt, M., Gillespie, M. A., Kunin, W. E. and McKerchar, M. (2022) Does agri-environment scheme participation in England increase pollinator populations and crop pollination services? *Agriculture, Ecosystems & Environment,* 325, 107755. <https://doi.org/10.1016/j.agee.2021.107755>.

Nowakowski, M. and Pywell, R. (2016) *Habitat Creation and Management for Pollinators.* UK Centre for Ecology & Hydrology. Available at: <https://www.ceh.ac.uk/sites/default/files/Habitat%20Management%20and%20Creation%20For%20Pollinators.pdf>.

Ouvrard, P., Transon, J. and Jacquemart, A. L. (2018) Flower-strip agri-environment schemes provide diverse and valuable summer flower resources for pollinating insects. *Biodiversity and Conservation,* 27(9), 2193-2216. <https://doi.org/10.1007/s10531-018-1531-0>.

Pebesma, E. (2023a) *sf: Simple Features for R*. Available at: <https://cran.r-project.org/web/packages/sf/index.html>.

Pebesma, E. (2023b) *lwgeom: Bindings to Selected ‘liblwgeom’ Functions for Simple Features*. Available at: <https://cran.r-project.org/web/packages/lwgeom/index.html>.

R Core Team (2023) *R: A language and environment for statistical computing.* R Foundation for Statistical Computing. Available at: <https://www.r-project.org>.

Redhead, J. W., Powney, G. D., Woodcock, B. A. and Pywell, R. F. (2020) Effects of future agricultural change scenarios on beneficial insects. *Journal of Environmental Management,* 265, 110550. <https://doi.org/10.1016/j.jenvman.2020.110550>.

Scholefield, P. A., Morton, R. D., Rowland, C. S., Henrys, P. A., Howard, D. C. and Norton, L. R. (2016) *Woody linear features framework, Great Britain*. UK Centre for Ecology & Hydrology. Available at: <https://www.data.gov.uk/dataset/eeb331d7-5000-4a93-8b4a-31621dd35cf4/woody-linear-features-framework-great-britain-v-1-0>.

UK Government (2023a) *Countryside Stewardship Grant Finder*. Rural Payments Agency and Natural England. Available at: <https://www.gov.uk/countryside-stewardship-grants>.

UK Government (2023b) *Environmental Improvement Plan 2023*. Department for Environment, Food and Rural Affairs. Available at: <https://assets.publishing.service.gov.uk/government/uploads/system/uploads/attachment_data/file/1168372/environmental-improvement-plan-2023.pdf>.

UK Government (2007) Hedgerow Survey Handbook: A standard procedure for local surveys in the UK. Department for Environment, Food and Rural Affairs. Available at: <https://www.hedgelink.org.uk/cms/cms_content/files/89_hedgerow-survey-handbook.pdf>.

Upcott, E. V., Henrys, P. A., Redhead, J. W., Jarvis, S. G. and Pywell, R. F. (2023) A new approach to characterising and predicting crop rotations using national-scale annual crop maps. *Science of the Total Environment,* 860, 160471. <https://doi.org/10.1016/j.scitotenv.2022.160471>.
